# Dynamic Absorption Enhancement and Equivalent Resonant Circuit Modeling of Tunable Graphene-Metal Hybrid Antenna

**DOI:** 10.3390/s20113187

**Published:** 2020-06-04

**Authors:** Zaka Ullah, Illani Nawi, Gunawan Witjaksono, Nelson Tansu, Muhammad Irfan Khattak, Muhammad Junaid, Muhammad Aadil Siddiqui, Saeed Ahmed Magsi

**Affiliations:** 1 Department of Electrical and Electronic Engineering, Universiti Teknologi PETRONAS, Perak 32610, Malaysia; illani.nawi@utp.edu.my (I.N.); muhammad_17000796@utp.edu.my (M.J.); muhammad_18003606@utp.edu.my (M.A.S.); saeed_19001716@utp.edu.my (S.A.M.); 2 Department of Information Technology, BRI Institute of Technology & Business, Jakarta 12550, Indonesia; gunawan.witjaksono@bri-institute.ac.id; 3 Center for Photonics and Nanoelectronics, Department of Electrical and Computer Engineering, Lehigh University, 7 Asa Drive, Bethlehem, PA 18015, USA; tansu@lehigh.edu; 4 Department of Electrical Communication Engineering, University of Engineering and Technology, Peshawar 25120, Pakistan; m.i.khattak@uetpeshawar.edu.pk

**Keywords:** plasmonic, graphene, terahertz, optical antenna, surface plasmons, tunability

## Abstract

Plasmonic antennas are attractive optical components of the optoelectronic devices, operating in the far-infrared regime for sensing and imaging applications. However, low optical absorption hinders its potential applications, and their performance is limited due to fixed resonance frequency. In this article, a novel gate tunable graphene-metal hybrid plasmonic antenna with stacking configuration is proposed and investigated to achieve tunable performance over a broad range of frequencies with enhanced absorption characteristics. The hybrid graphene-metal antenna geometry is built up with a hexagon radiator that is supported by the Al_2_O_3_ insulator layer and graphene reflector. This stacked structure is deposited in the high resistive Si wafer substrate, and the hexagon radiator itself is a sandwich structure, which is composed of gold hexagon structure and two multilayer graphene stacks. The proposed antenna characteristics i.e., tunability of frequency, the efficiency corresponding to characteristics modes, and the tuning of absorption spectra, are evaluated by full-wave numerical simulations. Besides, the unity absorption peak that was realized through the proposed geometry is sensitive to the incident angle of TM-polarized incidence waves, which can flexibly shift the maxima of the absorption peak from 30 THz to 34 THz. Finally, an equivalent resonant circuit model for the investigated antenna based on the simulations results is designed to validate the antenna performance. Parametric analysis of the proposed antenna is carried out through altering the geometric parameters and graphene parameters in the Computer Simulation Technology (CST) studio. This clearly shows that the proposed antenna has a resonance frequency at 33 THz when the graphene sheet Fermi energy is increased to 0.3 eV by applying electrostatic gate voltage. The good agreement of the simulation and equivalent circuit model results makes the graphene-metal antenna suitable for the realization of far-infrared sensing and imaging device containing graphene antenna with enhanced performance.

## 1. Introduction

Plasmonic innovation has appeared to be an essential field for exploring the potential outcomes of future photonics technologies. Various new research platforms have emerged after the investigation of its possible applications, including biosensing and imaging, biotech, astronomy, security, remote sensing, and optical communication [1]. Among a variety of photonic devices, plasmonic antennas are of great importance, having the ability to convert free space propagating electromagnetic radiation to highly localized energy, resulting in significant near-field enhancement [2]. Enhanced near-field empowers a wide verity of applications, such as spectroscopy, biosensing, light trapping, energy harvesting, and improved quantum emission [3]. Various modeling prerequisites, i.e., surface plasmon resonant wavelength, polarization, and spectral responses, should be considered in designing efficient plasmonic antennas. The vast majority of problems in optical antenna configuration are experienced primarily due to the air-dielectric interface. Consequently, the antenna will acquire reduced efficiency because of the trapped surface wave excitation [4]. Typical plasmonic antennas are recognized while using noble metals, such as silver and gold. The behavior of these materials at the infrared (IR) region changes because of finite electron mass in metals, which can lead to a delayed response to the incident EM field having a varying frequency [5]. Thereby, these metals act as lossy non-plasmonic conductors with the cutoff plasmon resonance frequency being oticed appropriately below the IR region, resulting in a considerable drop in metal conductivity [6]. This will undoubtedly give rise to propagation losses and attenuation, which also degrade optical antenna performance. Nevertheless, metallic plasmons endure problems with two major disadvantages: metallic plasmons are hardly tunable when the geometry of the plasmonic antenna is fixed and plasmon resonance frequencies are restricted to visible ranges [7].

The ongoing curiosity in the graphene plasmonics opens up the prospects to counterpart these impairments. Graphene is an allotrope of carbon, which has been revealed in recent years, having a monoatomic thickness and being purposely used as the nanomaterial in different fields [8]. Carbon atoms in the graphene sheet are arranged in the hexagonal lattice by forming a periodic honeycomb structure. Single-layer graphene is characterized by high carrier mobility that surpasses 20,000 cm^2^/V·s and 108 cm/s Fermi velocity at low temperatures [9]. The electronic band structure of graphene is the main reason for the extraordinary properties, i.e., better thermal coefficient, high mechanical strength, and excellent electrical properties. Graphene plasmonic properties are closely linked to its complex surface conductivity [10]. The tunability of graphene conductivity mainly depends on the Fermi energy, i.e., (chemical potential). Hence, altering the Fermi energy by applying electrostatic gate voltage across the graphene sheet or chemical doping significantly results in the tuning of graphene conductivity [11]. Alternatively, graphene plasmonic antennas have tunable abilities, despite having a fixed geometrical design. Moreover, graphene plasmons are sustained at terahertz (0.1–10 THz) and the whole infrared region, which is suitable for various probable plasmonic utilizations. Specifically, graphene nanoantennas are ideal for biosensing applications, as the biological molecules exhibit vibrational frequencies in the infrared region [12]. The extreme confinement of graphene plasmons results in the reduction of graphene antennas sizes to 1–3 orders smaller than the magnitude of free space propagating wavelength. The excellent tunability and miniaturization of graphene antennas render them extremely capable of sensing and imaging applications at infrared frequencies [13].

In recent years, cutting-edge nanofabrication technologies have empowered substantial improvement in developing optical antennas that are intended for tunable optoelectronic and photonic devices [14]. Graphene has been a promising candidate for tunable absorption handling and reconfigurability of resonant wavelength over a broad range of optical frequencies [15]. The dielectric permittivity and conductivity of graphene at optical frequency ranges are dynamically tunable, through the modulation of the carrier density under gate-controlled electrostatic bias, by permitting and prohibiting the generation and propagation of plasmons in the graphene sheet [16]. Thus, the tunable conductivity of graphene has been widely utilized for nanoscale devices in optical and terahertz regimes. Graphene plasmons display the utmost confinement of light, which allows for the graphene antennas to be designed at incredibly small (micro/nano) length-scales, while still effectively interacting with much longer wavelengths of light [17]. Specifically, absorption strength in graphene is one of the most crucial measures for control over photon collection. The assumptive schemes [18,19] have provocatively forecasted 100% (i.e., unity) absorption in resonantly excited graphene nanoantennas [20], demonstrating robust light-matter interactions in atomically thin graphene sheets.

A significant obstacle for accomplishing perfect absorption in fabricated graphene antennas is lower carrier mobility despite the stimulating forecasts and alluring features of graphene tunable antennas. Similarly, the low carrier mobility is due to polymethyl methacrylate (PMMA) residues in processed graphene samples or trapped impurities during the synthesis process. However, in theoretical works, the unpatterned and passivated graphene sheets with higher carrier mobility are adopted for predicting unity absorption [19,21]. The low graphene transport mobility results in a high non-radiative damping rate, whereby it tends to under-couple the resonant modes of graphene antennas in array design [22]. Subsequently, high-performance graphene-based efficient devices cannot depend on the graphene being processed through the exfoliation process [23]. However, the high-performance level is realized with graphene being synthesized through chemical vapor deposition (CVD) [21]. Furthermore, the effective carrier mobilities of graphene degrade when graphene plasmons are excited in patterned graphene resonator structures having edge defects and large carrier densities [24]. To avoid such problems, radiation coupling between graphene resonators should be increased, which is usually weakly credited to the thinness of graphene and mainly because of significant large wave-vector mismatch of free space propagating photons with excited graphene plasmons [25]. Until this point, various techniques, including deliberately designed substrates, huge chemical doping, and integration of graphene with noble metallic plasmonic antennas, have been investigated to boost the coupling of radiation to graphene nanoresonators [18]. Disregarding these endeavors, the reasonable complication in graphene resonator structures have experimentally constrained the optimum achievable absorption in graphene, and not even 50% of absorption has been recognized at infrared wavelengths [26].

An effective way to achieve broadband tunable absorption enhancement is by integrating graphene with metallic plasmonic antennas. Feng et al. [27] use the concept of light trapping and demonstrated substantial absorption enhancement, as the metallic plasmons improve the interaction with graphene plasmons at the resonant frequency. Recently, graphene metamaterial is employed to achieve enhanced broadband absorption at the mid-infrared range, approximately 85% absorption is experimentally demonstrated with 90 nm graphene thickness [24]. Yu et al., described the effect of stacked graphene dielectric structures [26], where the stacking of graphene creates plasmonic bandgap. Thereby, unity absorption is realized along with tuning at a broad range of frequency by changing the Fermi level of graphene. Efficient electrical tuning of optical absorption in a graphene-metal hybrid plasmonic device is demonstrated by the resonant coupling of plasmons between gold/graphene interface, and the tunability of strong absorption is acquired through changing the chemical potential by electrostatic gating [28]. In Ref [21], the graphene nanodisk array is integrated with an active device, and experimentally enhanced the absorption efficiency through voltage tuning. The absorption efficiency that was attained at the infrared region is tuned from 3% to 30% with tightly packed graphene nanodisks. Furthermore, various studies are reported by utilizing different graphene nanostructures to enhance the absorption along with enhancement of electric field [23,25,29]. Gric et al. [30], use the approach of photo-conductive antennas that is based on the optimized plasmonic nanostructure to numerically study the absorption enhancement in nanocylinders, where the absorption amplituide and resonant wavelength can be affected by the thickness and separation of nano cylinders. Additionally, absorption enhancement in the metal-dielectric-graphene sandwich structure is investigated while using unstructured graphene sheets [31], where the multiple order Fabery-Perot resonance results in multiband absorption [32].

The ultra-thin and small geometry of graphene antennas is highly dispersive with frequency-dependent properties. There is no accurate formulation and conventional hypothetical models for developing the connection among the graphene antenna performance and various related antenna characteristics. The approach based on full-wave numerical modeling is a method for characterizing the antenna performance. Still, with monoatomic thickness, it will surely require huge computing resources in the event when high accuracy is required during simulation [33]. The typical simulation method is quite complicated and it will hardly validate the basic theory principle of resonant antenna functionalities. A model-based parametric estimation (MBPE) is conducted on high impedance graphene nanotube antenna (CNT) to analyze antenna performance [10]. The resonant circuit models need to be developed to extend the knowledge on graphene antennas and to validate the simulations results to reveal insight into the parametric functionality of graphene antenna along with the physical understanding of resonant properties. Zhang et al. [34] proposed a simple RLC circuit model to explain antenna characteristics, i.e., resonance frequency and quality factor. For this purpose, they first consider the antenna input impedance with a series RLC circuit and treat the antenna as a coupled-resonator to optimize the RLC circuit values for resonance condition. In 2014, a transmission line that was based simple circuit model was proposed for plasmonic graphene dipole to calculate the input impedance and efficiency from the surface plasmon polaritons (SPPs) dispersion relation [35]. In another study, a very complicated partial element equivalent circuit (PEEC) technique is investigated for developing an equivalent circuit model for graphene antennas [36]. In [37], the authors employ the PEEC method to analyze radiated power and couplings. While, in [38], an equivalent circuit model is adopted to analyze graphene metamaterial absorber performance at the terahertz range.

In this article, a novel graphene-metal hybrid stack antenna is demonstrated to achieve enhanced absorption by designing graphene stacks with gold hexagon structure in a stacking manner. The proposed graphene antenna has tunable operational bandwidth, starting from 30 to 34 THz. The stacking symmetry provides a slow-wave structure, in which an efficient interaction of graphene plasmons takes place with the metallic plasmons in gold structure. The investigated antenna possesses tunable resonance behavior at a broad range of frequencies due to graphene tunable conductivity. The conductivity of the graphene layers in the stack is tuned by increasing the Fermi energy of the graphene sheet upon applying electrostatic gate voltage. The detailed study and operational mechanisms of the graphene-metal antenna are carried out through the parametric optimization of geometric and graphene parameters, which illustrate the tunable resonance behavior along with improvement in the overall performance. Additionally, the antenna performance and efficiency are also intensively investigated with different TM -modes excitation. The absorption enhancement is attributed to the stacking geometry of the antenna. Furthermore, an equivalent circuit model that is based on PEEC and transmission line method is modeled for the graphene patch and graphene-metal hybrid plasmonic antenna to validate the proposed geometric model. Good agreement is found between the numerical simulation results with the equivalent circuit model. Moreover, Section 2 discusses a theoretical illustration of graphene conductivity and permittivity, along with tunable conditions. Section 3 presents the details regarding the modeling of the graphene-metal stack antenna design. The equivalent circuit modeling is demonstrated in Section 4. While, in Section 5, the results and performance of the proposed model are analyzed, and the conclusion is drawn in Section 6.

## 2. Modeling of Graphene Surface Conductivity

The two-dimensional graphene material exhibits very outstanding electrical properties because of the mono-atomic thickness, where it allows for the propagation of surface plasmons modes at far-infrared frequencies. The surface conductivity model, electromagnetic wave propagation in single and multi-layer graphene, and also the effect of multi-layer graphene stacking on surface conductivity, along with the extraction of effective complex graphene permittivity, is investigated in this section. Kubo’s formula can be employed to calculate the surface conductivity of an infinite graphene sheet. The edge effects are not considered, which is experimentally proved in [39], stating that the edge effects of graphene conductivity only happen in designs possessing a lateral size of graphene smaller than 100 nm. Surface conductivity of graphene material is subjected to different attributes, i.e., Fermi level (µ_c_ = chemical potential), scattering rate (τ = relaxation time), frequency, and temperature. In particular, the electrical properties of graphene are dependent upon these parameters, with the change in these parameters producing a shift in surface conductivity due to the dispersive nature of graphene. According to Kubo’s formula [1], the surface conductivity of infinitesimally thin graphene surface with random phase approximation, i.e.,

(1) $$\begin{array}{l} \sigma \left(\omega ,\sigma ,\Gamma ,T\right)=\frac{jq^{2}(\omega -j2\Gamma )}{\pi \hbar^{2}}\\ \left[\frac{1}{{\left(\omega -j2\Gamma \right)}^{2}}\int_{0}^{\infty }\varepsilon \left(\frac{\partial fd(\varepsilon )-\partial fd(-\varepsilon )}{\partial \varepsilon }\right)\partial \varepsilon -\int_{0}^{\infty }\frac{fd(-\varepsilon )-fd(\varepsilon )}{{\left(\omega -j2\Gamma \right)}^{2}-4{\left(\varepsilon /\hbar \right)}^{2}}\partial \varepsilon \right] \end{array}$$

where *q* represents the electron charge, incident waves angular frequency is *ω*, *T* denotes the effective carrier temperature in kelvin, and *T* = 293 K, *ħ* = *h*/2*π* is the reduced Planck constant, Fermi–Dirac distribution is $f_{d}\left(\varepsilon \right)=\left(e^{\frac{\varepsilon -\mu c}{k_{B}}-T}+1\right)$, Γ = 1/2*τ* (relaxation time) denotes the collision frequency, and Boltzmann’s constant is represented by *k*_B_. The surface impedance of graphene is inversely proportional to conductivity and it can be considered by using boundary conditions *Z_s_* = 1/*σ_g_*. Moreover, the conductivity of graphene has two parts, intraband and interband transitions [3],

(2) $$\sigma_{g}=\sigma_{\mathrm{int}ra}+\sigma_{\mathrm{int}er}$$

In the infrared region, intraband contributions control the properties of graphene. If the intraband part of graphene conductivity is taken into consideration, and then in the absence of a magnetic field and eliminating the spatial dispersion effect. The surface conductivity of graphene can be approximated by the Drude model [6],

(3) $$\sigma_{\mathrm{int}ra}\left(\omega ,\mu_{c},\Gamma ,T\right)=j\frac{q^{2}k_{B}T}{\pi \hbar (\hbar \omega +j2\Gamma_{c})}\left[\frac{\mu_{c}}{k_{B}T}+2\ln(e^{-\frac{\mu_{c}}{k_{B}T}}+1)\right]$$

where *µ_c_* is the Fermi level (chemical potential) of the graphene sheet, damping constant is represented by *Γ_c_* = *q ħv_F_*^2^/*µµ_c_*, where the Fermi velocity is *v_F_* = 10^6^ m·s^−1^, and electron mobility is being denoted by *µ* = 10^4^ m^2^·V^−1^s^−1^. On the other hand, interband part of graphene conductivity dominates at high frequencies and it can be formulated as

(4) $$\sigma_{\mathrm{int}er}=j\frac{q^{2}}{4\pi \hbar }\ln\left[\frac{2\left|\mu_{c}\right|-\left(\hbar \omega +j\Gamma_{c}\right)}{2\left|\mu_{c}\right|+\left(\hbar \omega +j\Gamma_{c}\right)}\right]$$

### 2.1. Extraction of Multi-layer Graphene Stack Conductivity

It is worth pointing out that in this article, multi-layers of graphene in stack form are deposited on the top of the dielectric substrate, and a gold metal layer is to be sandwiched between two graphene stacks by forming hybrid graphene-metal antenna stack configuration. Each graphene stack is composed of three layers of graphene. Consequently, to calculate the conductivity of multi-layer graphene, it is assumed as the three layers of graphene were closely situated inside the stack. The effect of the dielectric substrate is strictly removed to find the conductivity of the tri-layer graphene stack. The approach of electrostatic gate bias [40] is being applied to investigate the properties of graphene stack, which regulates the carrier density in graphene layers, and afterward, the electromagnetic (EM) behavior of the graphene stack at the infrared region is analyzed. The proposed method is employed in two dependent stages. In the first stage, the carrier density is determined with respect to applied gate voltage while using an electrostatic bias approach. Whereas, in the second stage, the information that is obtained in the first stage is used to calculate the complex frequency-dependent conductivity of the stack. However, the carrier densities of tri-layer graphene biased with gate voltage can be approximated as

(5) $$qn_{s}^{top}=qn_{si}^{top}-C_{ox}^{top}V_{g}$$

(6) $$qn_{s}^{mid}=qn_{si}^{mid}+C_{ox}^{top}V_{g}+C_{ox}^{bot}V_{g}-C_{ox}^{mid}V_{g}$$

(7) $$qn_{s}^{bot}=qn_{si}^{bot}+C_{ox}^{top}V_{g}+C_{ox}^{mid}V_{g}-C_{ox}^{bot}V_{g}$$

where $n_{s}^{t,m,b}$ denotes the total carrier density of the graphene layers (*t,m,b* denotes top, middle, bottom layers), $n_{si}^{t,m,b}$. characterizes the pre-doping of graphene layers, and $C_{ox}^{t,\;m\;b}$ is the capacitance of the dielectric layer. When the values of carrier densities are known, the conductivity and chemical potential can be easily calculated to determine the EM behavior of the graphene stack. It is important to note that this electrostatic method estimated the graphene sheets as an infinite ideal conductor to calculate the carrier densities. Merging Equations (5)–(7), the chemical potential of each graphene layer can be found through [34],

(8) $$n_{s}=\frac{2}{\pi \hbar^{2}v_{F}^{2}}\int_{0}^{\infty }\varepsilon [fd(\varepsilon -\mu_{c})-fd(\varepsilon +\mu_{c})]d\varepsilon$$

Once the Fermi energies (*µ_c_*) are recognized for each of the graphene sheets, the complex conductivity of the graphene stack metal-hybrid structure can be extracted as

(9) $$\sigma_{s}(w)=i\sigma_{o}\frac{4E_{f,s}}{\pi }\frac{1}{\omega +i\gamma }$$

(10) $$\sigma (\omega )=\frac{e^{2}\hbar }{iS}\sum_{\alpha ,\beta }\frac{f(\varepsilon_{\alpha })-f(\varepsilon_{\beta })}{\varepsilon_{\alpha }-\varepsilon_{\beta }}\frac{\left|<\alpha \right|v_{g}{\left|\beta >\right|}^{2}}{\varepsilon_{\alpha }-\varepsilon_{\beta }+\hbar \omega +i\delta }$$

### 2.2. Effect of Chemical Potential on Graphene Conductivity & Permittivity

**Figure 1:**
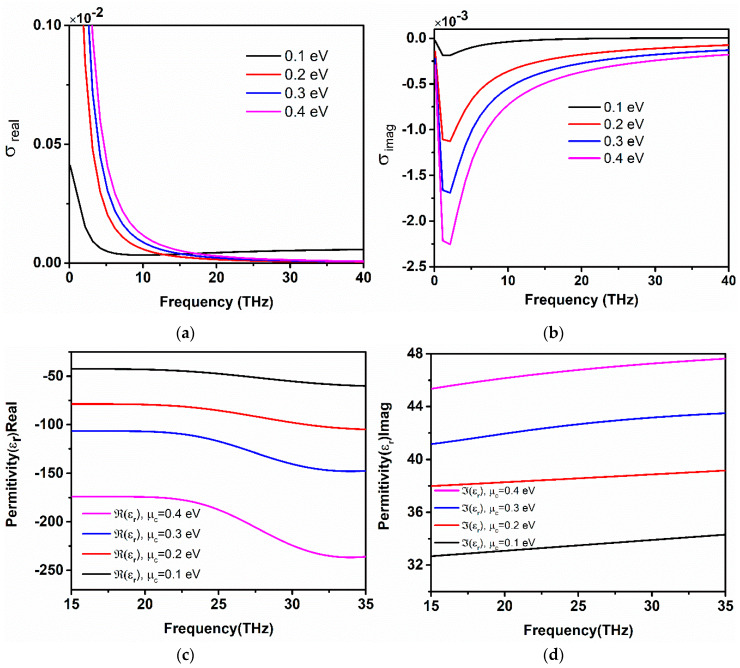
Complex graphene conductivity and permittivity with increasing chemical potential from 0.1 eV to 0.4 eV, (**a**) real part of graphene conductivity, (**b**) Imaginary part of graphene conductivity. (**c**) The real permittivity of multilayer graphene, and (**d**) Imaginary permittivity of multilayer graphene.

Chemical potential is a basic factor that can be progressively controlled by either chemical doping or electrostatic gate biasing. It should be noted that the chemical potential of the monolayer graphene depends on the carrier density *n_s_* of the film. Equation (8) correlates the carrier density and chemical potential of graphene. Contrarily, the carrier density is generally associated with the applied external electrostatic bias field. Consequently, the chemical potential is identified with applied biasing voltage through carrier density [34]. The gate bias is applied between the graphene stack and dielectric media to tune and actively control the conductivity [41]. In a graphene stack, each graphene layer act as a gate electrode. The Fermi level (chemical potential) and carrier density can be vigorously controlled in each layer graphene when the graphene layers are subjected to external gate bias [40]. The expression to understand the relation between *µ_c_* and *V_g_* is given by [42],

(11) $$E_{F}=\mu_{c}\approx \hbar v_{f}\sqrt{\frac{\pi \varepsilon_{r}\varepsilon_{o}V_{g}}{et}}$$

where $\varepsilon_{r}$ is the permittivity of the dielectric substrate, and *V_g_* is the bias voltage applied across graphene sheets. Figure 1a,b illustrate the real and imaginary part of graphene conductivity. Surface conductivity derived at *µ_c_* = 0.1 is low; however, as the chemical potential is raised to *µ_c_* = 0.4 eV, the conductivity obtained almost spans a wideband of frequency. Additionally, the increase in chemical potential provides tunable conductivity characteristics.

The thickness of graphene is supposed to be very thin, with the goal of getting the volumetric conductivity of graphene. Assuming dependence of time-harmonic on all EM fields, the equivalent complex permittivity can be derived from Maxwell’s equation $\nabla \times H=\left(\sigma_{g}-i\omega \varepsilon_{0}\right)\cdot E,$ where $\varepsilon_{0}$ represents free space permittivity. However, with the real and imaginary part, the equivalent complex permittivity of graphene layer with Δ-thickness can be expressed as $\varepsilon_{g}\approx -\sigma_{g,i}/\omega ∆+\varepsilon_{o}+i\cdot \sigma_{g,r}/\omega ∆.$ As the thickness of graphene Δ is monoatomic, neglecting $\varepsilon_{o}$ the real permittivity becomes $\varepsilon_{g,r}=-\sigma_{g,i}/\omega ∆.$ The relationship depicts that the real part of graphene permittivity has a negative sign that is contrasted with the imaginary part. The general equation for the permittivity of graphene can be derived from the conductivity by considering the incidence frequency and thickness of the graphene sheet [43].

(12) $$\varepsilon_{g}(\omega )=1+j\frac{\sigma_{g}(\omega )}{\omega \varepsilon_{0}\Delta }$$

Additionally, the real permittivity of graphene is negative at THz range can be observed in Figure 1c, while Figure 1d shows the imaginary part of graphene permittivity, which is positive. The effect of chemical potential on both the real and imaginary permittivity is analyzed. It is observed that increasing the chemical potential of graphene layers from 0.1 eV to 0.4 eV, the real part of permittivity becomes more negative, while the imaginary part remains positive for all the values of chemical potential. In Figure 1c, the dependence of frequency on the real part of permittivity is illustrated; at the fixed value of chemical potential, the permittivity drops to more negative when the frequency is increased. On the other side, Figure 1d depicts the imaginary permittivity rises slightly with an increase in frequency, while the value of chemical potential remains fixed.

## 3. Hybrid Graphene-Metal Antenna Designing Theory

**Figure 2:**
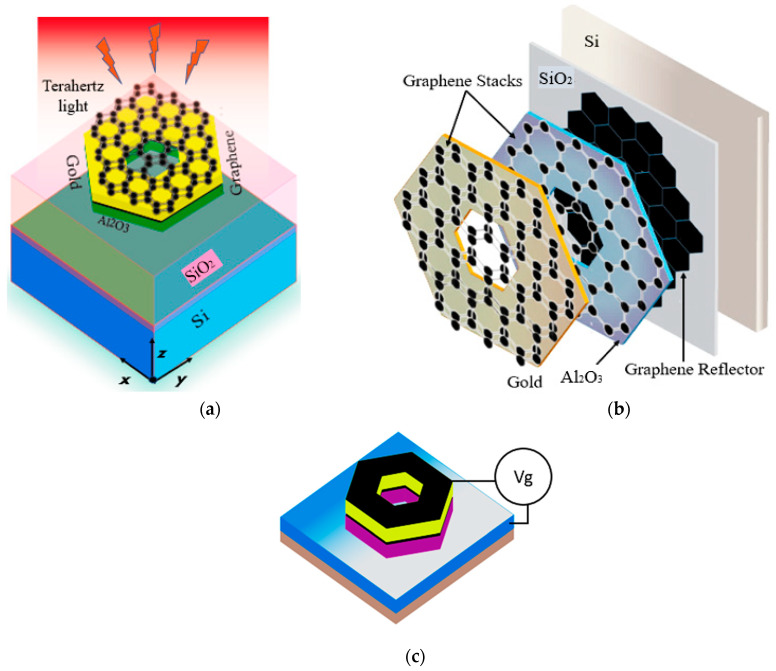
Proposed geometry of graphene-metal hybrid nanoantenna. (**a**) Top view of the graphene stack antenna, as the antenna is illuminated by THz light in the frequency range of 30–34 THz and is incident on the surface of antenna parallel to the z-axis, (**b**) layered view of the antenna structure, as the antenna structure is composed of eight-layers, i.e., three-layers of substrates, two graphene stacks, a gold layer, and graphene reflector, and (**c**) gating of the proposed antenna by applying a voltage across graphene sheets for the tuning of Fermi energy in the range of 0.1 eV–0.3 eV.

A graphene-based stack metal hybrid antenna is designed in the infrared region, specifically at 33 THz in the far-infrared region. Figure 2a illustrates the schematic diagram of the investigated graphene antenna configuration, where the graphene-metal antenna structure comprises of tri-layer graphene stack on the top of slotted gold hexagon shape, followed by second graphene stack, which is supported by Al_2_O_3_ insulator layer of the same dimensions as a gold layer. Finally, multi-layer graphene that is used as a back metal reflector has impinged on a silicon wafer having a thin SiO_2_ substrate layer with permittivity $\varepsilon_{r}$ = 3.9. The function of the reflector to prevent the leaking of radiations into the SiO_2_ /Si substrates. The bottom geometry of the graphene stacked antenna, which forms and supports the basic hybrid structure, is the silicon/silicon dioxide substrate having dimensions along *x* × *y*-axis of 15 × 15 um, while the thickness of Si 0.5 um and SiO_2_ thickness 0.3 um respectively. Usually, utilizing a thin substrate helps in the lessening of surface waves and dielectric losses. Subsequently, substrates with high dielectric constant provide better confinement of energy with low leakage losses. Although, the antenna efficiency, along with fractional bandwidth, decreases. The gold hexagon has a radius of 6 um with a center gap having a radius of 2 um, while the thickness of the gold layer is chosen as 0.4 um, as depicted in Figure 2a. However, there is a second graphene stack between the gold hexagon and Al_2_O_3_ hexagon. The thickness of the Al_2_O_3_ hexagon is set 0.1 um, working as an insulator layer [44] between the graphene back reflector and the second graphene stack. Although, the addition of the insulator layer (Al_2_O_3_) to the antenna geometry reduces the electrical (quantum) tunneling from the sandwich graphene-metal stack to the graphene reflector [45], and the same time this insulator layer protects the graphene reflector from the effect of metal doping from the graphene-metal sandwich [46]. The gold structure is sandwiched between two graphene stacks with the purpose of exciting the surface plasmon resonance (SPR) on the surface of graphene and to trap the local electric field to increase the interaction of incident infrared radiation with the graphene sheets. Thus, the graphene plasmons can be actively coupled to metallic plasmons of the gold layer by providing enhanced absorption, along with highly-confined electric fields at the center gap of hexagon radiator. When the top surface of a gold resonator is loaded with graphene stack, the localized electric field at the edges of gold hexagon drives the graphene plasmons, which starts flowing along the x-direction, resulting in highly enhanced energy confinement at the center gap of the gold resonator. The graphene sheet placed on the SiO_2_ substrate functions as a reflector, where the absorption of the stacked antenna structure can be expressed as $A=1-{\left|S_{11}\right|}^{2}$, as $S_{11}$ denotes the reflection coefficient of the antenna.

The structure is simulated through a finite-difference time-domain (FDTD) computational solver in CST studio to hypothetically investigate the optical characteristics of the proposed design. A linearly polarized transverse magnetic (TM) plane wave (the electric field is propagating along the z-axis). The incidence waves have the angular polarization ‘θ’ in the x-axis direction with a frequency range of 28–34 THz, in order to excite the graphene-metal antenna structure. In the simulation setup, the x-axis boundary is set to a perfect electric conductor, while a perfect magnetic conductor boundary is applied along the y-axis. The thickness of graphene is set to ≈1 nm, because the stack is composed of three graphene layers. To get better performance, the characteristics parameters of graphene are optimized by setting the chemical potential value as 0.3 eV, relaxation time as 0.1 ps, and temperature *T* = 300 k, respectively. Furthermore, increasing the surface conductivity of graphene by applying gate voltage across graphene stack results in the high interaction of metallic plasmons with graphene plasmons, which in turn increases the absorption in the graphene-metal antenna.

The dimensions of the designed antenna model are 15 × 15 um, but to perform parametric study and analyze the effect of various geometric parameters, i.e., (changing the radius of hexagon and center gap, substrate width, the addition of graphene layers, and increasing gold thickness) on the performances of the antenna are systematically studied. Larger absorption is achievable by employing multilayer graphene with metallic and dielectric stackings. The proposed graphene-metal antenna has unique and exceptional functionalities; which, firstly, it can highly confine the electric field and enhance absorption, and secondly, it provides the tunability of surface plasmon resonance at a wide range of far-infrared frequneices. Figure 2b depicts the gating of the antenna where the external bias voltage is applied to vary the Fermi energy (0.1 to 0.4 eV) for the tuning of graphene surface conductivity. Moreover, the key characteristic of a graphene-metal antenna is surface plasmon resonance, and this property of the graphene nanoantenna can be tuned to any frequency of interest by changing the chemical potential of the graphene layers. The tunable behavior of plasmon resonance with increasing Fermi energies can be related under the condition, which states that the wave vector *k_spp_* propagating along the hexagon is related by *k_spp_* = *n*/*R*, where *R* is the radius of the hexagon and *n* is the mode order. In the considered frequency range, the surface plasmon in graphene approximately satisfies $k_{spp}=\hbar \omega^{2}/\left(2\alpha E_{F}c\right)$, where $\alpha =e^{2}/\hbar c$ represents the fine structure constant. Consequently, the resonance frequency of surface plasmons can be formulated as

(13) $$\omega_{spp}\approx \sqrt{2n\alpha E_{F}/\hbar R}\propto \sqrt{nE_{F}/R}$$

Equation (13) delivers a rough estimation of surface plasmon resonance and the tuning of resonance frequency with Fermi energy instead of varying the geometrical size of the graphene antenna. This property of graphene antenna makes it more useful then metallic optical antennas. Similarly, the plasmon resonance of nanoantennas can be firmly affected by using different dielectric substrates. Accordingly, in the proposed investigation, the graphene-metal antenna is designed on the stacked substrate, thereby surface plasmon resonance happens at

(14) $$\hbar \omega_{spp}\approx {\left(\frac{2\alpha \hbar cLE_{F}}{\pi (\varepsilon_{1}+\varepsilon_{2}+\varepsilon_{3})D}\right)}^{\frac{1}{2}}$$

where *L* represents the length, *D* denotes the diameter of the proposed antenna, and $\varepsilon_{1}$, $\varepsilon_{2}$, $\varepsilon_{3}$ is the dielectric permittivities of silicon, silicon dioxide, and alumina oxide, respectively.

The broadly utilized coupled oscillator model [47,48] is adopted to analyze the absorption spectra and near field interaction of the resonances between the stacked layers due to the effect of graphene coupling in an effort to elucidate the physical mechanism of the graphene-metal stacked antenna frequency tunability behavior. The effect of coupling between the two resonances is related to change form the radiative state to the dark plasmonic state. The sandwich structure at the top of antenna geometry that consists of graphene-metal-graphene is considered as oscillator 1, and the graphene stack in between the Al_2_O_3_ layer and SiO_2_ is represented by oscillator 2. The charges in *χ*_1_(*t*) in oscillator 1 can be legitimately excited by the incident radiation source, and the charges *χ*_2_(*t*) can only be excited by the near field coupling of oscillator 1, they fulfill the following coupled equations [49],

(15) $${\ddot{\chi }}_{1}(t)+\gamma_{1}{\dot{\chi }}_{1}(t)+\omega_{1}^{2}\chi_{1}(t)+k^{2}\chi_{2}(t)=gE$$

(16) $${\ddot{\chi }}_{2}(t)+\gamma_{2}{\dot{\chi }}_{2}(t)+{\left(\omega_{2}+\delta \right)}^{2}\chi_{2}(t)+k^{2}\chi_{1}(t)=0$$

where *γ*_1_ and *γ*_2_ are the damping rates in the oscillators 1 and 2, while *ω*_1_ and *ω*_2_ are the angular frequencies of the resonance modes in both oscillators, respectively. δ describes the detuning of resonance frequency from the dark plasmonic state to the radiative state. *k* represents the coupling coefficient between the dark plasmonic mode and radiative plasmonic modes. At the same time, g is the geometric parameter that represents the strength of coupling between the radiative plasmonic mode with the incident infrared radiation. Solving Equations (15) and (16), the susceptibility for the proposed antenna model can be expressed, as [50],

(17) $$\chi_{e}=\frac{g\times (-\omega^{2}+i\gamma_{2}\omega +{\left(\omega_{2}+\delta \right)}^{2}}{(-\omega^{2}+i\gamma_{1}\omega +\omega_{1}^{2})\times (-\omega^{2}+i\gamma_{2}\omega +{\left(\omega_{2}+\delta \right)}^{2})-k^{2}}$$

The absorbance of the stacked graphene-metal antenna configuration can be described as

(18) $$A=1-T=1-\left|\frac{c(1+n_{s})}{c(1+n_{s})-i\chi_{e}}\right|,$$

Finally, the Lorentz profile for the absorbance at a resonant frequency can be directly calculated from the dispersion relation for the coupled Lorentz oscillator model [51]; the absorbance at the angular resonance frequency ω of the proposed structure is,

(19) $$A(\omega )=\frac{2\pi \chi_{e}d}{\underset{A_{o}}{\underbrace{\gamma_{a}\ln10}}}\frac{{\left(\gamma_{1}/2\right)}^{2}}{{\left(\omega_{1}-\omega_{2}\right)}^{2}{\left(\gamma_{2}/2\right)}^{2}}$$

where *d* is the thickness of the stacked profile, *γ_a_* is the total absorption rate, while *A_o_* is directly related to oscillator strength. The transmission of the second oscillator should be blocked (*γ*_2_ = 0) and the mode damping rate *γ*_1_ should be equal to the absorption rate *γ_a_* in order to get unit absorption at the resonance frequency (*ω*), thus satisfying the critical coupling condition.

## 4. Equivalent Circuit Modeling of Graphene-Metal Stack Antenna

The graphene-metal antenna that was investigated in this article resonates at a broad range of frequencies (i.e., 30–34 THz) as graphene exhibits metal-like properties at the infrared region. Normally, various complex numerical and theoretical techniques are used to study the resonance behavior, performance, and validate the graphene antenna simulation results. For this purpose, an equivalent circuit model for the graphene-metal stacked antenna is developed. First, a simple graphene patch is analyzed while using a circuit model based on the partial element equivalent circuit (PEEC) technique to determine the resistance and inductance of graphene sheets. Second, the unit cell PEEC model is considered for a stack graphene hexagon patch to determine the resonant frequency. Lastly, the RLC equivalent circuit model is developed for the graphene-metal hybrid antenna to study the performance characteristics and resonance behavior.

### 4.1. Circuit Model of Graphene Hexagone

A PEEC method is employed in order to understand the surface resistance and inductive behavior of graphene stack at the top of gold hexagon functioning as a graphene radiator. The proposed PEEC method states that a normal graphene patch is composed of the addition of many PEEC cells. The cells are either rectangular or triangular for the graphene patch. Here, the hexagon symmetry is divided into rectangular cells, which is the most convenient way, and several systematic solutions exist for the equivalent circuit calculation [36]. The PEEC method is based on the electric field integral equation (EFIE) to derive a unit cell equivalent circuit for graphene. The total electric field is the sum of incident and scattered field when the graphene sheet is excited by the THz source, which is

(20) $$E_{total}=E_{inc}+E_{Sca}=E_{inc}-\frac{\partial A}{\partial t}-\nabla \phi =\frac{J}{\sigma }$$

However, the above equation relates to the tangential electric field and surface electric current through the surface conductivity of graphene. The impedance of the graphene sheet can be directly determined from the surface conductivity by considering impedance boundary condition at *z* = 0, and it can be formulated as

(21) $$\hat{n}\times (H_{|z=0+}-H_{|z=0+})=J_{surf}=\sigma (\omega )E$$

$J_{surf}$ is the current density of graphene hexagon surface and σ(ω) is the total surface conductivity of graphene containing intraband and interband transitions. The surface resistance of a rectangular graphene patch having a length (*l*) and width (*w*) can be expressed by applying the PEEC model on one rectangular unit cell, being expressed by

(22) $$R_{surf}=\frac{1}{w\sigma (\omega )}=R_{r}+J\omega L_{r}+Z_{\mathrm{int}er}$$

**Figure 3:**
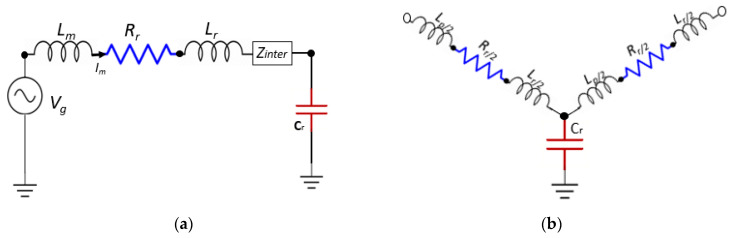
(**a**) Partial element equivalent circuit (PEEC) rectangular cell circuit model for graphene patch, where Vg represents the external bias voltage due to applied external electric field, Lm denotes the self-inductance, Rr, Lr and Zinter is due to graphene complex surface conductivity; and, (**b**) Unit cell model for the stacked graphene, where the two branches share the same node.

$R_{surf}$ is the total impedance due to graphene conductivity, $R_{r}$ and $L_{r}$ represent the resistance and inductance from the interband part of graphene complex conductivity, while $Z_{inter}$ is the impedance resulting from the interband part. The operating frequency of the proposed antenna in the far-infrared region, particularly at 33 THz, where the total surface conductivity is dominated by intraband contribution and the behavior of graphene conductivity is also dispersive. The developed PEEC is well-matched to the graphene patch, and the resistive effect of the circuit model represents the loss of graphene due to the real part of conductivity, as depicted in Figure 3. However, the inductive and capacitive effect in a circuit is because of the imaginary part of graphene conductivity. The imaginary part of graphene conductivity can accomplish negative values, as indicated by [52]. Consequently, the derived equivalent circuit components reactive responses are neglected by the imaginary part. Hence, applying voltage *V_g_* at the unit cell PEEC equivalent circuit and neglecting the interband contribution of surface conductivity. The total surface resistance can be calculated, as

(23) $$R_{surf}=\frac{1}{w\sigma_{\mathrm{int}ra}(\omega )}=R_{r}+J\omega L_{r}$$

The $R_{r}$ denotes physical resistor and corresponds to the real part, while ω is proportional to the imaginary part and it relates to the $L_{r}$ physically pure inductor. Finally, each component in Equation (23) can be written in terms of resistance and inductance as

(24) $$R_{r}=\frac{l}{w}\frac{\tau^{-1}}{\frac{2e^{2}k_{B}T}{\pi \hbar^{2}}\ln\left[2\cosh\left(\frac{\mu_{c}}{2k_{B}T}\right)\right]}$$

(25) $$L_{r}=\frac{l}{w}\frac{1}{\frac{2e^{2}k_{B}T}{\pi \hbar^{2}}\ln\left[2\cosh\left(\frac{\mu_{c}}{2k_{B}T}\right)\right]}$$

From Equation (25), it is clear that, when intraband conductivity is considered, the reactance in Equation (23) is always positive, while $R_{r}$ in Equation (24) is the ohmic losses in the conducting graphene. The absorption cross-section is directly proportional to branch current (Im) for the graphene patch unit cell circuit model, as seen in Figure 3. The absolute value of the branch current Im can be estimated by solving the circuit model, and the values of branch current attain maximum value at certain frequency points known as the resonant frequency.

### 4.2. Resonant Frequency Calculation of Graphene Hexagone Based on PEEC Circuit Model

Surface plasmon resonant frequency for the graphene hexagon stack shape patch can be straightly calculated from the subsequent equivalent circuit model. Figure 3b illustrates the partial equivalent circuit for two current threads, which share a common node point on the graphene hexagon due to PEEC partition. However, both of the two branches are a simplified version of the unit cell equivalent circuit, as in Figure 3a. For resonant frequency calculation, some simplifications are applied in the development of the circuit presented in Figure 3b, i.e., the interband transition is neglected, because low infrared frequencies (far-infrared) are considered for the operation of graphene hexagon. Next, the coupling between the two branches is omitted, because the surface plasmon polaritons (SPPs) are excited due to the collective resonance of electrons. Henceforth, in this model, the effect of voltage control sources and mutual inductances are not considered. Lastly, a uniform source is considered along the orthogonal direction to graphene hexagon, and a factor of 1/2 is applied to the RLC components sharing the same node to further simplify the proposed model. Therefore, each graphene sheet can be separately modeled with the proposed PEEC model. Although the proposed model is somewhat similar to the transmission line (TL) model [53], and also there exist some differences between the PEEC model and the TL model. The branch impedance and admittance can be derived for the above model based on these assumptions as,

(26) $$Z^{\prime }(\omega )=R^{\prime }+j\omega {L^{\prime }}_{r}+j\omega {L^{\prime }}_{p}=R^{\prime }+j\omega L^{\prime }$$

(27) $$Y^{\prime }(\omega )=j\omega C^{\prime }$$

where the Equations (26) and (27) represents the impedance and admittance of the equivalent circuit for graphene hexagon. The total inductance is represented by $L^{\prime }=L/p$, where *L* = *L_p_* + *L_r_*, $C^{\prime }=C/p$ is the capacitance and *p* denotes the unit length of a single cell. Additionally, the propagation constant in both branches of the circuit model can be related to impedance and admittance by

(28) $$\gamma =\alpha +j\beta =\sqrt{Z^{\prime }Y^{\prime }}$$

Thus, the dispersion relation of the graphene hexagon patch can be determined as,

(29) $$\omega^{2}=\frac{{\left(\beta p\right)}^{4}}{LC{\left(\beta p\right)}^{2}+R^{2}C^{2}/4}$$

The dispersion relation of the graphene presented by Equation (29) can be reduced to the ordinary TL model dispersion relation as, $\beta p=\sqrt{LC}$, when the resistance R = 0 is considered in Equation (29). Applying the Fabry–Perot type resonator condition on graphene hexagon having an effective length, Equation (29) becomes

(30) $$l_{eff}=m\frac{\pi }{B}$$

where *l_eff_* is the effective length of the resonator and m is the integer. Finally, excluding losses i.e., (*R* = 0) and combining Equations (29) and (30) determines the resonant frequency for graphene hexagon through the proposed circuit model.

(31) $$\omega =\frac{mp}{l_{eff}}\frac{\pi }{\sqrt{LC}}$$

### 4.3. Equivalent RLC Resonant Circuit Model of Graphene-Metal Stacked Antenna

**Figure 4:**
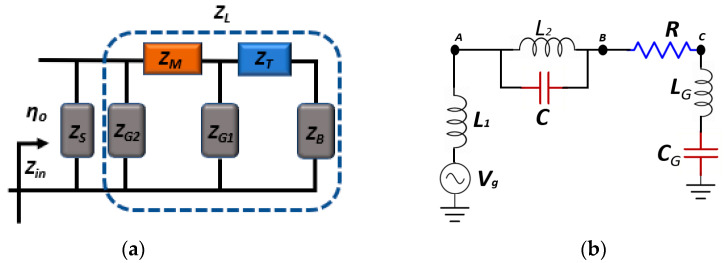
(**a**) Transmission line model of the graphene-metal antenna; (**b**) Equivalent RLC resonant circuit model of the hybrid antenna derived from the corresponding transmission line model.

In this section, the transmission line (TL) model and the corresponding RLC resonant equivalent circuit model are developed to analyze the performance of the designed antenna, respectively, in Figure 4a,b. Assuming the proposed graphene antenna as a coupled-resonator, the functioning frequency of the antenna is the resonance frequency of the proposed RLC equivalent circuit, as depicted in Figure 4b. The input impedance ($Z_{in}$) governs the reflection and transmission coefficients of the antenna, according to TL theory. The Input impedance must be perfectly matched to the characteristic impedance ($Z_{s}$) to obtain the resonance frequency of the circuit model. Input impedance $Z_{in}$ is acquired with

(32) $$Z_{in}=\frac{(R_{s}+jX_{s})jX_{l}}{(R_{s}+jX_{s})+jX_{l}}$$

where $Z_{L}=jX_{L}$ is the whole impedance of the graphene-metal stacked structure. In Figure 4a, the input impedance of the graphene back reflector, and graphene stacks is $Z_{B}=Z_{G1}=Z_{G2}=\frac{1+j\omega \tau }{\sigma }\cdot Z_{M}=j\left(\omega L-1/\omega C\right)Z_{c}$ denotes the impedance of coupled gold resonator between the graphene stacks. $Z_{T}$ is the impedance of the incorporated Alumina oxide (Al_2_O_3_) between the graphene reflector and the first graphene stack, which can be expressed as $Z_{T}=jZ_{s}\tan\left(\gamma l\right)$, where γ is the wavenumber $\gamma =\frac{\omega \sqrt{\varepsilon_{r}}}{c}$ and *l* is the thickness of the Al_2_O_3_, respectively. Similarly, the reflection coefficient (Γ) can be determined from the input impedance, as,

(33) $$\Gamma =\frac{Z_{in}-\eta_{o}}{Z_{in}+\eta_{o}}$$

The resonance frequency of the proposed RLC equivalent circuit can be formulated as,

(34) $$\omega_{o}=\frac{1}{\sqrt{LC}}$$

From Equation (34), it is clear that the resonance frequency of the circuit is dependent on the values of L and C. Furthermore, the quality factor is studied for the proposed RLC circuit, which has an inverse relationship to antenna bandwidth. Accordingly, unloaded quality factor $Q_{a}$ and coupled quality factor $Q_{o}$ are borrowed to describe the resonance performance of the proposed antenna. Hence, quality factor for the coupled resonator antenna and coupling coefficient can be related to the resonance frequency

(35) $$Q_{o}=\frac{\omega_{o}L}{R},\beta =Z_{o}/R$$

where *L* is the inductance, *R* is the resistance corresponding to the resonance frequency of the circuit, and β is the coupling coefficient. Normally, as indicated by [34], from the Reflection coefficient (*S*_11_) frequency response, the quality factor at a random point in the frequency range can be expressed by the following equation

(36) $$Q_{a}=(f_{o}/2){\left|f_{a}-f_{o}\right|}^{-1}$$

From Equation (36), it can be seen that the quality factor is directly related to the bandwidth of the antenna at the resonance frequency $f_{a}$. Additionally, the quality factor depends on the selected points in the resonance frequency, if a random point selected far away from the resonant point, then the resonance quality factor can be determined by

(37) $$Q_{0}=aQ_{a}$$

The correction coefficient ‘*a*’ value at the frequency ‘*f_a_*’ with reflection magnitude can be calculated as

(38) $$a={\left[\frac{{\left(1+\beta \right)}^{2}\left|S_{11}{\left(fa\right)}^{2}\right|-{\left(1-\beta \right)}^{2}}{1-\left|S_{11}{\left(fa\right)}^{2}\right|}\right]}^{1/2}$$

## 5. Simulation Results and Discussion

**Figure 5:**
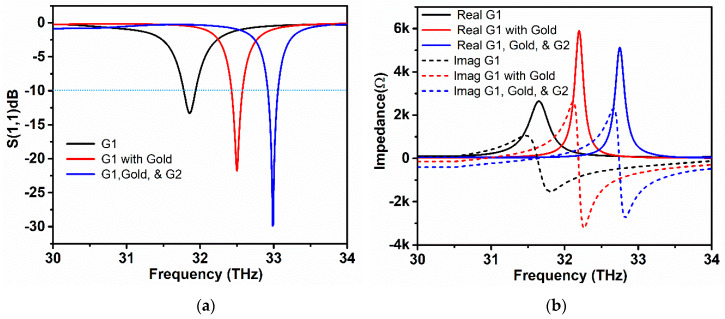
(**a**) Reflection coefficient of the proposed graphene-metal antenna and the effect of stacking symmetry on the *S*_11_; and, (**b**) Input impedance Real and Imaginary part corresponding to the resonance frequencies of stacking symmetry.

The graphene-metal plasmonic antenna has been excited with plane wave having a frequency range of 30–34 THz, in investigating the characteristic performance of the proposed graphene-metal stack antenna. The parametric optimization of the graphene and the corresponding frequency responses are obtained while using Finite-difference time-domain (FDTD) solver in the CST studio. In achieving better results, the mesh grid in numerical simulations is set to the minimum cell size of 0.02 um, therefore, generating a fine mesh across the geometry. Furthermore, in the RLC equivalent circuit that is illustrated in Figure 5b, the parameters are extracted by designing and optimizing the circuit using the software package AWR design environment. Additionally, the investigation is carried out by altering the L and C values in the proposed circuit to analyze their effect on the resonance frequency and quality factor. The simulation results obtained from the CST studio are compared with the RLC circuit to validate the proposed model.

The multilayer graphene complex conductivity that is dependent on the frequency is calculated through the Kubo formula, as depicted in Figure 1a,b, and realized in the simulation as an impedance boundary condition in CST studio. The tuning range of chemical potential is considered from 0 eV to 0.3 eV to evaluate the frequency tunability response. However, the relaxation time is set to 0.3 ps along with temperature 300 K for the graphene stacks. The proposed graphene-based antenna is tuned to 33 THz having the chemical potential of 0.3 eV and it has potential applications in terahertz sensing, infrared imaging, and spectroscopy.

### 5.1. Surface Plasmon Resonance

The surface plasmon resonance of the proposed simulated graphene-metal stack antenna is described by the reflection coefficient (*S*_11_), as shown in Figure 5. It can be clearly observed that the proposed antenna with two graphene stacks and a gold layer sandwich between them resonates at 33 THz having *S*_11_ value of −30 dB, as represented by the blue line in the Figure 5a, below. Here, the effect of the graphene stack sandwich structure is also compared with the resonance behavior of one graphene stack without the gold layer. The simple graphene stack resonates at 31.8 THz with a value of −14 dB, which is clear that only graphene-based patch antenna having a mismatch at 31.8 THz. However, when the gold hexagon layer is placed in the top of the first graphene stack, the resonance frequency shifts rightwards in the frequency range and displays an enhancement of *S*_11_ value −22.5 dB. The shifting of frequency is due to the effective coupling between graphene plasmons and metallic plasmons. This effect is noticeable at high frequencies ranges, which also provide the frequency tunability feature to metallic nanoantennas. The chemical potential and relaxation time of the graphene stacks is set to 0.3 eV, and 0.3 ps, while the thickness of the gold hexagon layer is 0.4 um, while the thickness of the Al_2_O_3_ separation layer is 0.5 um. Finally, placing the second graphene stack on the top of the gold layer completes the hybrid geometry of the proposed antenna, and the structure becomes a slab of repeating substrate, metal, and graphene stacks, as illustrated in Figure 2b. Through the addition of the gold layer and second graphene stack, the reflection value increases because of better impedance matching of the structure with free-space radiation. The antenna has −10 dB bandwidth of 0.3 THz, which remains the same as the resonance point shifts to higher frequencies.

Figure 5b illustrates the real and imaginary impedance of the proposed antenna at the resonance point for the above-explained symmetries. Input impedance is the characteristic of an antenna, which provides a clear understanding of how well the designed antenna is matched with incident frequency. The solid lines depicted in Figure 5b represent the real part of the input impedance, while dashed lines depict the imaginary part of the input impedance, respectively. The input impedance with only one graphene stack is quite low when compared with graphene/gold and graphene/gold/graphene symmetry at the resonance frequency. The input impedance of the final stack configuration is lower than the graphene/gold symmetry due to the contact resistance effect of the graphene stacks with a gold layer. At 33 THz, the input impedance has a value of 5.2 K ohms by providing a better matching condition between the incident EM fields and the antenna. An excellent agreement between the reflection coefficient and impedance spectra can be observed from Figure 5a,b, which agree with the matching condition.

### 5.2. Effect of Chemical Potential On Resonance Frequency

**Figure 6:**
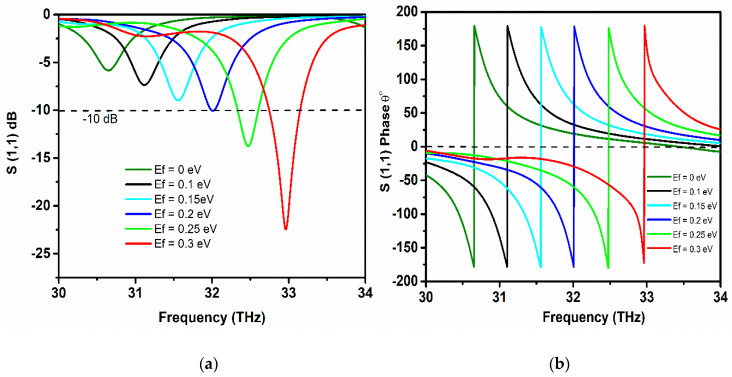

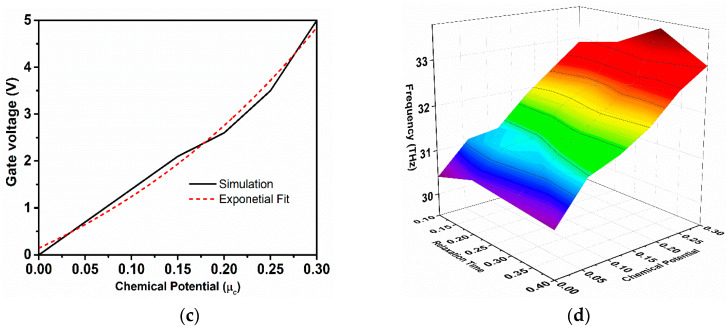
(**a**) Reflection coefficient of the proposed graphene-metal antenna with varying chemical potential ranging from 0 eV to 0.3 eV; (**b**) Reflection phases of *S*_11_ with varying chemical potential; (**c**) Tuning of chemical potential with respect to varying gate voltage; and, (**d**) three-dimensional (3D) graph of resonance frequency tuning with respect to chemical potential and relaxation time.

Chemical potential, which is also known as Fermi level (*E_F_*), plays a significant role in controlling the complex surface conductivity of graphene. The chemical potential of the graphene sheets is tuned in the range of 0 eV to 0.3 eV by applying external electrostatic gate voltage across the graphene sheet, as shown in Figure 2c. In this subsection, the resonance shift that is based on increasing the chemical potential with a step width of 0.05 eV to observe the resonance tuning and to achieve two times enhancement in operating bandwidth by setting the relaxation time 0.3 ps. The resonance frequency tuning is realized by varying gate voltage, which in turn increases the chemical potential of the graphene layers. The significant increase in the chemical potential tune the surface conductivity of graphene to higher frequencies, therefore, providing the tunability at a wide range of frequencies, starting from 30.6 THz to 33 THz. Figure 6a illustrates the resonance tunability of the proposed graphene-metal antenna with varying chemical potential. The reflection coefficient at 0 eV is less then –10 dB and the antenna resonates at 30.6 THz. However, the increasing chemical potential up to 0.15 eV the *S*_11_ is improved along with frequency tuning. At 32 THz, the resonance peak touches the −10 dB line, which ensures the antenna matching condition. This dramatic right shift of the frequency is directly related to the chemical potential of graphene layers in stacks. Additionally, the *S*_11_ resonance peaks accomplish better values as the chemical potential raises. With the chemical potential of 0.3 eV, the antenna attains the resonance peak with *S*_11_ of −23 dB at 33 THz. It should be noted that the value of *S*_11_ at 0.3 eV in Figure 6a is less than the values attained in Figure 5a because the thickness of the substrate between the graphene first graphene stack and graphene reflector is varied to 1um. The effect of fringing fields between the graphene stack and metal reflector is minimized due to increasing substrate thickness, thus resulting in the improvement of bandwidth. The -10dB bandwidth at 0.3 eV with 1 um substrate thickness is 0.6 THz, which is almost double than the previous one with a substrate thickness of 0.5 um, respectively.

Figure 6b represents the respective phases of *S*_11_ at the resonance frequencies with varying chemical potential, as the resonance frequency shift rightwards, the phase of *S*_11_ also tune to the resonance position. Figure 6c depicts the tuning response of the chemical potential with increasing gate voltage, which undoubtedly depicts that varying gate voltage linearly increases the chemical potential of graphene. The black line represents the calculated chemical potential values with variable gate voltage from the simulation, while the red dash line is the exponential fitting of the calculated values gate voltage versus chemical potential. Furthermore, the effect of varying chemical potential with increasing, relaxation time on the resonance frequency can be examined from Figure 6d. It can be observed that, when the chemical potential value of graphene layers remains constant and the relaxation time is varied from 0.1 ps to 0.4 ps, a very small shift of resonance frequency is observed. However, a better impedance matching condition is reflected when the relaxation time is set 0.4 ps with a high value of *S*_11_. In contrast, when chemical potential is varied, the graph shows an inclined behaviour of the resonance frequency. Thus, the tunability of the proposed antenna mainly depends upon the chemical potential of the graphene layers.

### 5.3. Frequency Response of the Antenna with Respect to Parametric Optimization

**Figure 7:**
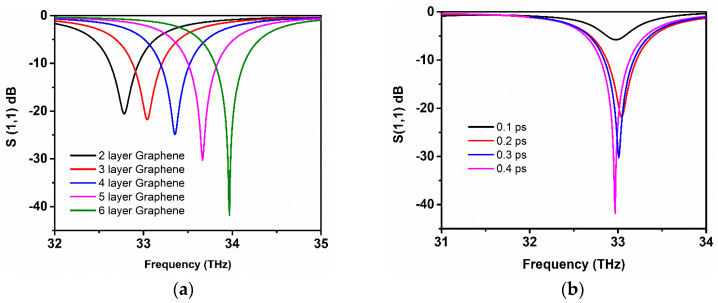
(**a**) Resonant frequency tuning with increasing graphene layers in the stack; and, (**b**) Effect of the relaxation time on the reflection coefficient.

The performance of the designed graphene-metal antenna is investigated with respect to the antenna geometric parameters and other graphene properties. Firstly, the *S*_11_ parameter and resonance frequency are studied. A dramatic shift of resonance towards higher frequencies with increased *S*_11_ values were observed as the number of graphene layers in a stack is increased to six layers of graphene. The highest resonance peak is attained at 34 THz with *S*_11_ of −42 dB when the stacks of the antenna are composed of six layers of graphene, as shown in Figure 7a. However, increasing graphene layers can affect the radiation efficiency and EM wave confinement due to the low coupling of graphene plasmons with metallic plasmons and the increase of interlayer resistance between the graphene sheets. Although, the addition of more layers can significantly increase the absorption of the antenna along with the tuning of the resonance frequency. In Figure 7b, the behaviour of *S*_11_ parameter; the relaxation time, which is known as the scattering rate of the graphene, has been observed to increase. This property of graphene improves the resonance peak by achieving higher reflection values while the frequency remains static. At minimal value, the relaxation time decreases the *S*_11_ peak. However, by increasing the value of relaxation time to 0.4 ps, the reflection peak accomplishes sharp resonance at 33 THz with *S*_11_ of almost −42 dB. As can be observed from Figure 7b, a higher value of relaxation time helps in improving the operational bandwidth of graphene antenna, but a very small shift can be observed in the frequency towards lower frequency. It should be noted that the graphene antenna that was investigated in this article has a relaxation time of 0.3 ps.

#### 5.3.1. Effect of Gold Hexagon Size and Thickness

**Figure 8:**
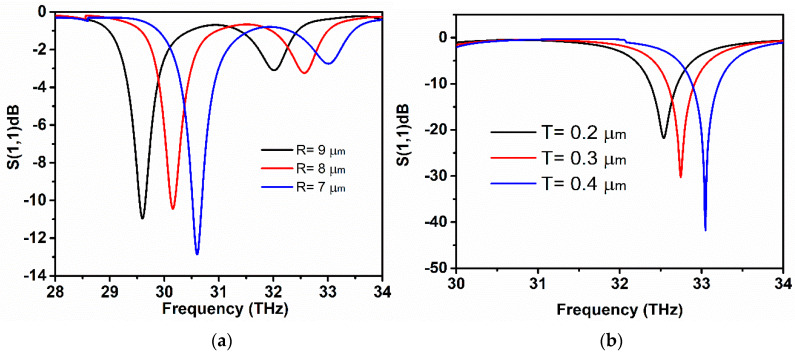
(**a**) *S*_11_ with respect to the increasing size of the gold hexagon; and, (**b**) Resonance tuning of the proposed antenna by decreasing the thickness of the metallic layer.

The metallic part of the antenna plays a significant role in the operation of the graphene antenna. Normally, metallic antennas have a broad range of operating frequencies due to the interaction with incoherent diffraction along the edges and corners. The surface plasmons dthat propogate along the edges and sharp corners of the metal patch can produce highly EM field confinement, which can be exploited in the application where near-field interaction is needed. The hexagon symmetry with a center slot is adopted to achieve better EM field confinement. It is challenging to attain tunable performance when metallic antenna geometry matched to fix frequency, as the conductivity of metals cannot be tuned like tunable graphene conductivity with an increasing chemical potential. The tunability of the metallic plasmonic hexagon resonator is possible by altering its size. The proposed antenna has a gold hexagon having a radius of 6 um with the center slot of 2 um in radius. The effect of antenna’s performance is analyzed by increasing the radius of the metallic hexagon. Figure 8a shows that, as the antenna’s size increases, the proposed antenna’s surface plasmon resonance resonates at lower frequencies. This validates the inverse relation of the antenna’s length and the resonating frequency. As the radius of a hexagon is set to 7 um, the antenna resonates at 30.6 THz, which is far lower than the target frequency, but also possesses dual-band operational resonance frequency. The dual-band resonance is realized when the size of the antenna is increased, and the inductance also rises, which in turn increases the electrical length, resulting in multiband resonance. Here, the antenna radius is varied from 0.7 um to 0.9 um, and the resonance frequency has shifted to 29.5 THz. The operating frequency shifts leftwards to lower frequencies as the size of the antenna becomes larger. However, the second band at higher frequency has low impedance matching because of the low *S*_11_ value. Figure 8b illustrates the resonance tuning of the antenna when the thickness of the gold layer is changed. The resonant length decreases when the thickness of the metallic layer is increased. Essentially, it could be explained by the interference of two group waves. One type directly reflects from the surface while the other is captured by the metallic cavity when the EM waves propagate on the metallic hexagon radiator patch. This type of wave results in the shifting of the resonance frequency. As the thickness increases, the phase difference between these two types of group waves also increases, which further results in the spilling of radiation fields from the metallic patch and, hence, increases the resonant length. Figure 8b clearly shows the effect of increasing metallic patch thickness. As a result of increasing resonant length, the antenna resonance shifts rightward to high frequencies.

#### 5.3.2. Effect of the Dielectric Substrate on the Performance of Graphene-Metal Antenna

The utilization of a dielectric substrate is apparent for the viability of the graphene antenna design and empowers innovative physical phenomena and working modes. An appropriate selection of substrates material can proficiently control different electromagnetic properties. The effects that are applied by the substrate on the performance of the antenna can be analyzed by increasing the dielectric constant or changing the substrate thickness. The conventional scaling rule states that resonance frequency, $f=\varepsilon_{r}^{-1/2}$ is inversely proportional to dielectric constant. Hence, with lower dielectric substrates, the antenna attains higher resonance frequencies and vice versa. However, predicting the resonance condition of the graphene-metal antenna structure with the above rule is a challenging task because the resonance field of the proposed antenna is an open boundary. The relative changes in physical and material parameters of antenna empower scaling of the antenna with fluctuating resonance frequency, as long as the losses and dispersion are weak in the used material. Additionally, partial scaling of the resonant position is conceivable by increasing the dielectric permittivity and thickness of the substrate, while also keeping the graphene antenna geometry fixed. Additionally, plasmon propagation of the graphene metal antenna exhibits interesting characteristics due to the increase of dielectric permittivity, resulting in the downscaling of frequency from the resonance position. Consequently, a numerical investigation is carried out in CST studio with increasing dielectric constant and substrate thickness to explore the influence of dielectric media on the surface plasmon resonance frequency.

**Figure 9:**
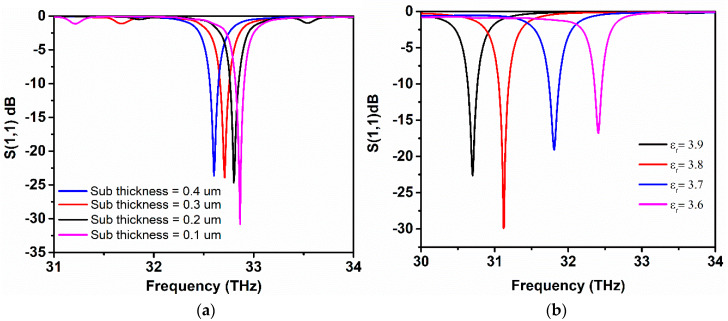
(**a**) Effect of the increasing substrate thickness on the resonance frequency; and, (**b**) The effect of increasing substrate permittivity on graphene antenna performance.

Figure 9a demonstrates the effect of changing substrate thickness on resonance frequency, as the thickness is varied, the resonance of antenna redshifts in the lower frequency range with a shift of 0.1 THz. The increase in the thickness of the graphene antenna substrate minimizes the fringing fields and induces EM radiation with better efficiency. However, the resonance peak moves leftwards with a decrease in *S*_11_ value with the increases in thickness. Figure 9b depicts the proposed antenna performance with respect to the change of the dielectric permittivity. The dielectric permittivity of the SiO_2_ substrate in the designed antenna is *ε_r_* = 3.5. The effect of increasing dielectric permittivity of substrate material on the frequency response of the graphene antenna is investigated through simulations. It is obvious from Figure 9a, that, by increasing the permittivity of the substrate, the resonance frequency has a redshift and similarly achieves sharp resonance at lower frequencies as the permittivity raises.

### 5.4. Characteristic Mode Analysis

In this section, characteristic mode analysis has been demonstrated to reveal the total and modal input impedance and efficiency of the proposed graphene-based antenna. The natural resonant capability of complex graphene-based structures is directly related to the linear superposition of decomposed modes of impedance and radiation efficiency. Multiple modes of the antenna resonance can be acquired by solving integral equations on the antenna geometry in a single simulation, which has the advantage of less computational requirements. Furthermore, the impedance matrix of graphene-based antennas is very dissimilar from that of optical metallic antennas. The desired substrate properties should be carefully taken into consideration, along with the lossy and reactive behavior of the dielectric substrate material. The characteristic modes of the graphene-based antenna can be calculated by resolving the eigenvalues for the impedance matrix [54] (Z = Zr + Zl), represented by

(39) $$XJ_{n}=\lambda_{n}RJ_{n}$$

where *J_n_* represents the associated eigenvector, *l_n_* is the eigenvalue, and *n* is the modal number, *X* = *X_r_* + *X_l_* is the imaginary part of the impedance *Z*. In Equation (34), the dissipated and radiated contributions are denoted by subscripts *l* and *r*. The dissipation impedance matrix *Zl* describes the complex surface conductivity of graphene. Similarly, the modal input admittance can be expressed with respect to the characteristic current [54], as follows

(40) $$Y_{n}=\frac{J_{n}(p)J_{n}(p)l_{p}l_{p}}{1+j\lambda_{n}}$$

From the above equation, the total input impedance can be calculated as *Z_in_* = 1/*Y_in_*. Additionally, the impedance of decomposed modes with various characteristic modes can be achieved by manipulating the graphene properties, which can provide further detailed insight into the antenna performance. Additionally, the radiation efficiency is a countable parameter of the graphene plasmonic antennas that is directly related to the ratio between total incident power on the antenna surface to the reflected power from the antenna. The relation of radiation efficiency can be found here by substituting the induced current with CM Eigen currents,

(41) $$\eta_{n}^{rad}=\frac{P_{n}^{rad}}{P_{n}^{rad}+P_{n}^{loss}}=\frac{J_{n}^{H}R_{r}J_{n}}{J_{n}^{H}(R_{r}+R_{l})J_{n}}$$

**Figure 10:**
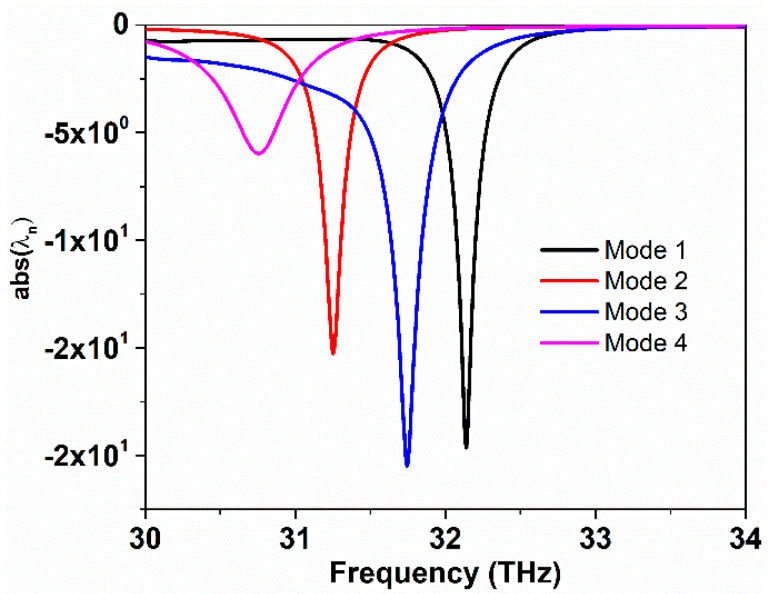
Resonance frequency response with respect to four different transverse magnetic (TM) Characteristic modes.

Four different modes are studied in order to analyze the characteristic modes of the proposed graphene-based antenna. The first and third modes are excited by TM polarized waves i.e., propagation vector normal to the antenna surface (x-polarized). While the second and fourth modes are excited with the same waves, but with the wave vector propagating parallel to the antenna surface (y-polarized). Figure 10 illustrates the characteristic mode analysis of the proposed graphene antenna with a relaxation time set to 0.4 ps. The odd modes are x-polarized and they have the resonance frequency higher than even modes. The excited four modes have resonance frequencies at 32.2, 31.2, 31.8, and 30.8 THz, respectively.

#### 5.4.1. Input Impedance Analysis with Characteristic Mode

**Figure 11:**
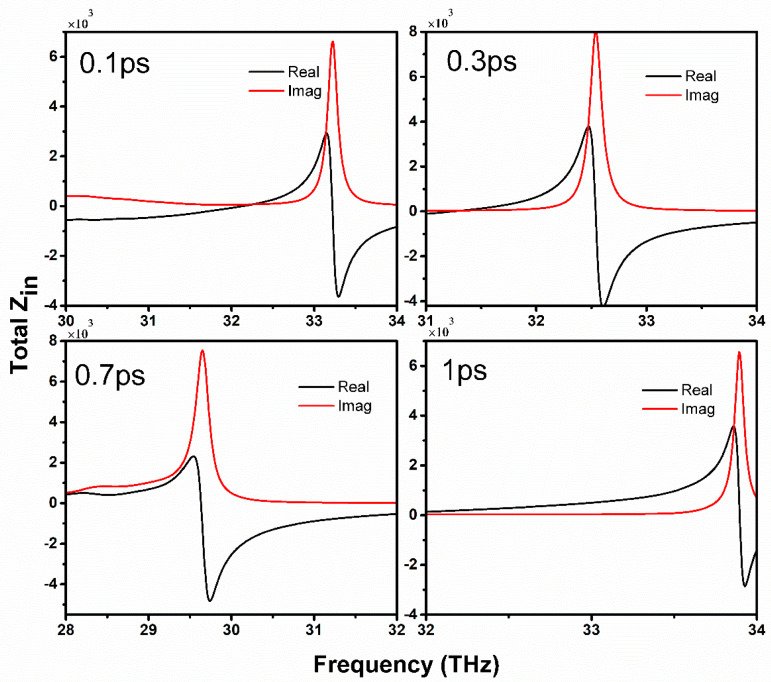
The real and imaginary input impedance at four different characteristic modes.

The total input impedance of the proposed antenna is investigated based on characteristic modes with different excitation modes. Figure 11 illustrates the total input impedance of the characteristic modes with varying relaxation times of 0.1, 0.3, 0.5, and 1 ps. The different relaxation times are selected to demonstrate the influence of graphene quality on resonance performance. The red lines represent the real part of the total impedance and the black lines are used to describe the imaginary part of the total input impedance. When the value of relaxation time is 0.1 ps, and the polarization of the incident wave is normal to the antenna surface, the antenna resonates 32.2 THz, where the input reactance is almost zero, because of extremely high input impedance. In this case, the resonance frequency is at 32.2 THz, while the impedance appears somewhat at a high frequency due to the superposition of different modes propagating on the dame surface. It can be noted that that graphene sheets having a low value of relaxation time 0.1 ps contribute less to the antenna resonance. Thus, the sharp resonance frequency peaks and the graphene quality can be improved by increasing the relaxation time.

Furthermore, at 0.3 ps the input impedance becomes more significant and the resonance of the antenna appears at 32.6 THz, because the wave vector is propagating parallel to the antenna surface. The higher input impedance is obtained at 0.7 ps, which is also in good agreement with resonance frequency with a small redshift. However, at mode 4, when the relaxation time is set to 1 ps, the antenna has zero reactance at the desired frequency, as shown in Figure 10. This is due to the non-appearance of the input impedance, which might be caused by the characteristic mode. Instead, it occurs at the high-frequency end caused by the superposition of mode 2 and mode 4.

#### 5.4.2. Modal Input Admittance and Radiation Efficiency

**Figure 12:**
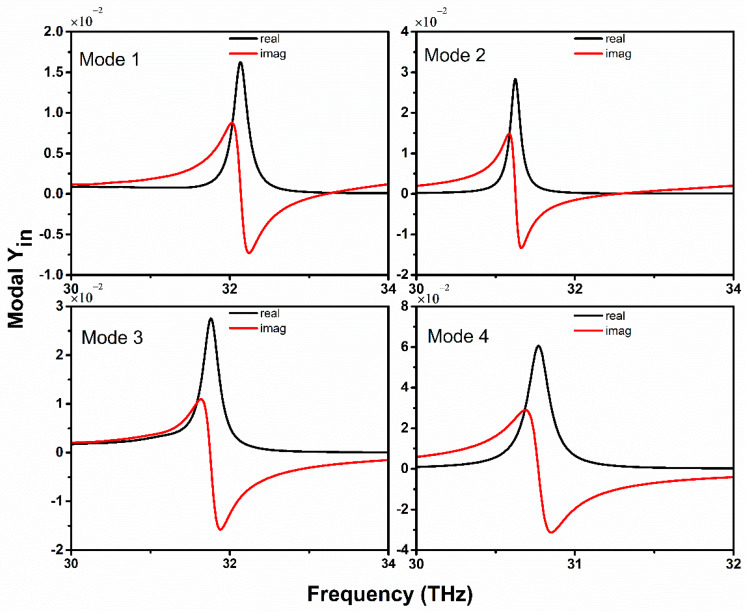
The real and imaginary input admittance at four different characteristic modes.

The modal input admittance outcomes from the simulation also expose the resonant condition of the proposed graphene-metal antenna. Figure 12 demonstrates the input admittance results with a different relaxation time of graphene and it is excited with four different modes. The black lines represent the real part of the modal input impedance, while the red lines indicate the imaginary part of modal input admittance. Additionally, the modal input admittance results are in good agreement with the resonant condition, as shown in Figure 10. Additionally, it can be observed from Figure 12, that mode 2 and 4 display a higher value of conductance relative to the odd modes and they also have the possibility to excite resonance modes. It can be noted that mode 1 has sufficiently low resistance value at the resonance frequency, which approves mode 1 as the fundamental mode of the antenna and can achieve lower resistance when the relaxation time is being increased.

**Figure 13:**
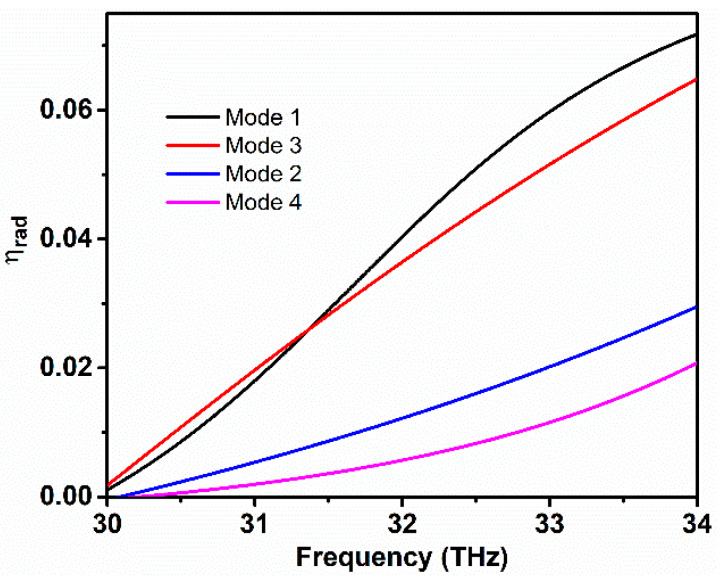
Radiation efficiency corresponding to characteristic modes.

Additionally, radiation efficiency analysis that is based on the characteristic mode method is analyzed since efficiency is the key factor of the graphene antenna. Figure 13 illustrates the modal efficiency of the graphene antenna with characteristic modes. The odd modes present a very dramatic rise in efficiency as the frequency becomes high. However, the efficiency that is depicted from the even modes is relatively less to odd modes and, also, up to 20% of the efficiency can be achieved when the frequency moves to a higher range. Mode 1 has shown high achievable frequency because of the low resistance at the resonance frequency.

### 5.5. Tunability and Enhancement of Optical Absorption

The optical absorption enhancement of graphene-based antennas, along with the tuning of optical absorption at the broad spectral range, particularly far-infrared, stays problematic and provides an extremely exhaustive task. The limitation of low optical absorption in the graphene-based antenna is overcome by employing multilayer graphene in stacking configuration in the antenna geometry. The proposed technique allows for the antenna to achieve higher absorption along with tunability at the broad spectral range, even at terahertz to mid-infrared. The graphene stack on the top of the gold hexagon has the advantage of exciting surface plasmons propagating modes, which results in high EM field confinement. Furthermore, the absorption of multilayer graphene is enhanced by sandwiching the gold hexagon between graphene stacks, which also allows for the propagation of plasmons and plasmonic light trapping to increase the absorption. The acquired unity absorption (i.e., 100%) is tunable in a band starting from 30 THz to 34 THz. The tunability is realized by applying gate voltage across the graphene reflector being separated by the first graphene stack with an insulator Al_2_O_3_ spacer. Using a substrate layer between the mirror and first graphene stack provides a wide range of tunable surface plasmons excitation in the graphene layers. When incident light strikes the top graphene stack, it then excites plasmons propagating modes along with the excitation of localized surface plasmons in the gold metallic structure. These localized surface plasmons trap the light energy in the near field, which leads to stronger EM confinement and enhanced absorption. The graphene surface that is below the gold layer can absorb some of the light from the interaction of graphene and gold plasmons and it can achieve unity absorption. The interband absorption of the stacked graphene can be greatly enhanced by the effect of metal doping and the increase of the Fermi energy with bias voltage. In the proposed stacked structure, the resonance *S*_11_ is totally caused by the graphene-metal sandwich, if the total absorption of the antenna is designed at frequency $f=v2E_{F}/(2\pi \hbar c$), *S*_11_ can be greatly enhanced by increasing the *E_F_* through the electrostatic doping [55]. This tunability from the low absorption to unity absorption is quite important for the realization of infrared sensors. Finally, the conductivity model of the doped graphene (electrostatically raising the graphene Fermi energy) to express the absorption enhancement by local-RPA model [55],

(42) $$\sigma (\omega )=\frac{e^{2}}{4\hbar }\frac{1}{\left(\omega +i\tau^{-1}\right)}\left[E_{F}^{T}-\int_{0}^{\infty }dE\frac{f_{E}-f_{-E}}{1-4E^{2}/\left[\hbar^{2}{\left(\omega +i\tau^{-1}\right)}^{2}\right]}\right]$$

where $E_{F}^{T}=E_{F}+2K_{B}Tlog\left(1+e^{-E_{F}/K_{B}T}\right)$ represents the thermal correction at the doping level, while $f_{E}=1/\left[1+e^{\left(E-E_{F}\right)/K_{B}T}\right]$ is the Fermi-Dirac distribution. Probably, the absorption is greatly enhanced from 35% to 100% by increasing the Fermi energy by 0.3 eV at 33 THz. However, in a realistic device, the doping of graphene is tuned by the relative potential difference [32], i.e., applying voltage V. In this case, *E_F_* = 0.3 eV is obtained through a potential of 5 V.

**Figure 14:**
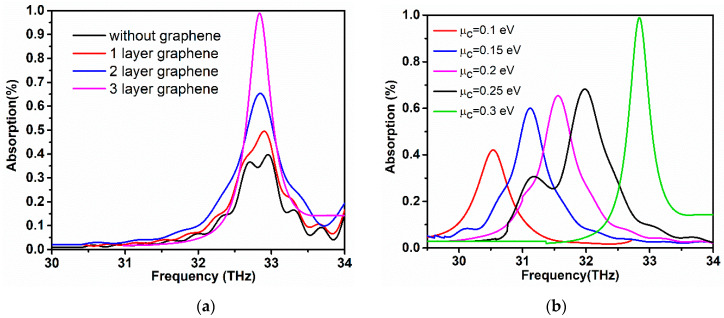

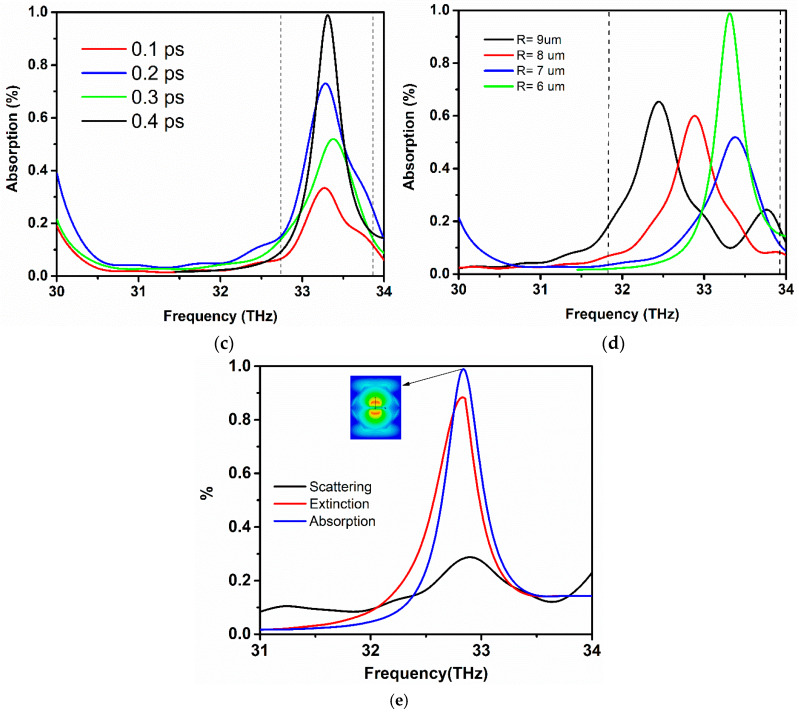
(**a**) Absorption spectra with graphene layers, (**b**) Absorption spectra tuning with chemical potential, (**c**) Effect of relaxation time on absorption, (**d**) Tuning of absorption spectra by increasing hexagon size, and, (**e**) Comparison of the simulated absorption, Scattering and extinction of the antenna at the resonance frequency, i.e., 33 THz with the graphene Fermi energy tuned to 0.3 eV. The black line represents the scattering magnitude, which is far lower than the absorption magnitude, thus validating the proposed structure has the ability to absorbs the maximum portion of the incident light.

Figure 14a represents the dramatic absorption of the proposed graphene-metal antenna under the normal excitation being illuminated by TM plane wave with normal incidence to antenna surface. It is observed from Figure 14a that the optical antenna without graphene exhibits a deficient optical absorption with a maximum peak of 35%. The absorption spectra are analyzed with several graphene layers, with the antenna with monolayer graphene being placed on the hexagon resonator attaining the absorption of almost 50%. As the layers of graphene are increased, the absorption rises sharply and achieves almost unity absorption at the resonance frequency. In the proposed design, graphene stack with three layers acquired unity absorption spectra, while bi-layer graphene displays 65% of absorption. In comparison to the single-layer graphene-metal antenna, the multi-layer graphene-metal antenna hybrid structure provides a high modulation of optical absorption characteristics and strong enhanced absorption. Figure 14b represents the dynamic tunability of absorption spectra when the electrostatic gating raises the chemical potential of the graphene layer. It can be observed that, at the low chemical potential, the antenna exhibits low optical absorption. However, as the chemical potential is increased, the absorption spectra show inclined behavior, reaching almost 100% when the chemical potential reaches 0.3 eV. The tuning range of absorption spectra starts from 30 THz to 34 THz. At higher chemical potential, the metallic gold structure serves as a light-trapping component that increases the interaction of graphene with incident light, therefore increasing the strong absorption. It is worth noting that the substrate layer that is used between the graphene reflector and graphene first stack has a significant effect on the absorption spectrum. Additionally, the graphene reflector also plays a significant role in achieving a complete absorption by the constructive interference of the reflection from the hexagon stack and reflector. Strong absorption can be guaranteed with a low tuning range, and can actively tune the phase of the reflected light.

Figure 14c shows the absorption spectra related to the increment of graphene relaxation time. Surprisingly, the absorption becomes higher as the relaxation time is increased. Additionally, the absorption spectra are less tunable when relaxation time is set to a higher value. At 0.1 ps, 30% of absorption is achieved by the antenna; however, at 0.4 ps, the absorption spectrum reaches to maximum, i.e., 100%. On the other hand, the lower relaxation time shifts the resonance spectrum to a higher frequency, with the absorption peak being centered at 33.4 THz. The optical absorption of the graphene-metal antenna can be controlled and tuned by varying the gold hexagon size. Figure 14d shows the absorption spectra that were related to the increasing radius of the metallic hexagon. The redshift of absorbance spectra occurs primarily because of the increasing size. However, the peak of absorption spectra drops with a larger size, and the absorption bandwidth has shown increment. The metallic part in graphene antennas can produce small tunability but it can significantly decrease the absorption at the absorption resonance relatively to match the graphene-metal antenna structure. Moreover, to acknowledge the stacked structure being responsible for the unity absorption at the resonance frequency (33 THz), the absorption spectra are compared with the stacked antenna extinction and scattering responses. Figure 14e illustrates the comparison of the absorption, extinction, and scattering simulated response under the normal incidence of the incident light. It can be observed that the stacked geometry largely absorbs the incoming incident light. However, a small fraction of light results in scattering, which is relatively far lower than the magnitude of absorption.

#### 5.5.1. Unity Absorption Tunability and Electric Field Distribution

**Figure 15:**
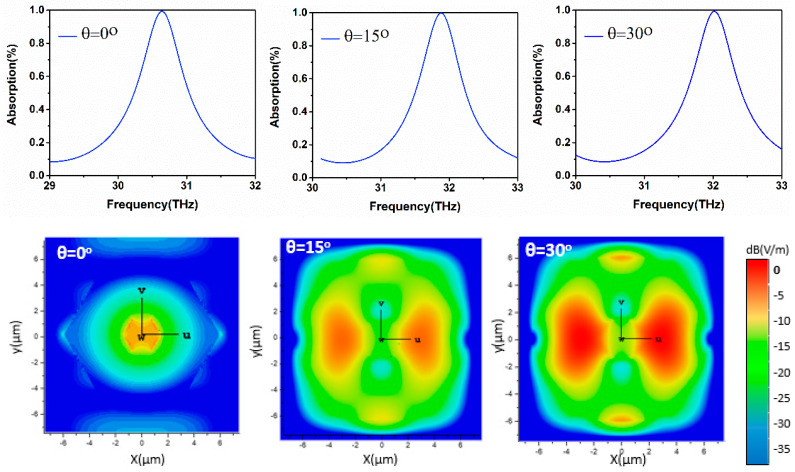
Absorption tuning and the corresponding E-field distribution to incidence angles 0°–30°.

The effect of absorption and electric field distribution of the designed antenna with respect to the incidence angle of the incident light source is analyzed in order to demonstrate the tunability of unity absorption and field distribution. The incidence angle of the source light is tuned in the range of 0° to 90°, which can actively tune the absorption characteristics along with the enhancement of highly EM confine fields at the center of the antenna design. The unity absorption realized from the stacking structure of the antenna design under the normal incidence of the light source. The incident light source is TM y-polarized having electric field vector, which propagates normal to the antenna surface. Once the unity absorption that is achieved with the stacking structure under the normal incidence of the light source with TM y-polarized waves with electric filed propagating vector normal to antenna surface, then the angle of incidence of the y-polarized wave is changed to investigate the tunable absorption and electric field distribution due to surface plasmons propagation. First, the angle of incidence is set to θ = 0^°^, having propagating vector parallel antenna geometry. The unity absorption resonance occurs at 30.5 THz, as the incidence angle greatly affects the tunability of absorption resonance with respect to normal incidence, as shown in Figure 15. At θ = 0°, the electric field distribution emerges at the center gap of the antenna due to the excitation of surface plasmon resonance and starts propagation from the center gap towards the whole hexagon geometry.

On the other hand, the density of the electric field is quite minimum. When the incidence angle is set to θ = 15°, the absorption resonance shows blueshifts across the frequency range with 0.4 THz, along with the maximum electric field at the sides of the hexagon structure. The electric field is dense at the horizontal edges due to propagation surface plasmons originating from the sharp corners of the hexagon, but the density of the electric field is minimum at the center gap of the antenna, and the EM fields are less confined. However, the fields show better confinement at the horizontal edges when the incidence angle is set to θ = 30°. Here, the surface plasmons start to propagate from the edges of the hexagon to the center of the antenna, as depicted in Figure 15. Although the absorption peak shifts to 32.1 THz, selecting an incidence angle between 0° to 30° can provide the tuning of optical absorption from 30.4 THz to 32.1 THz with less confined EM fields at the center gap of the antenna.

**Figure 16:**
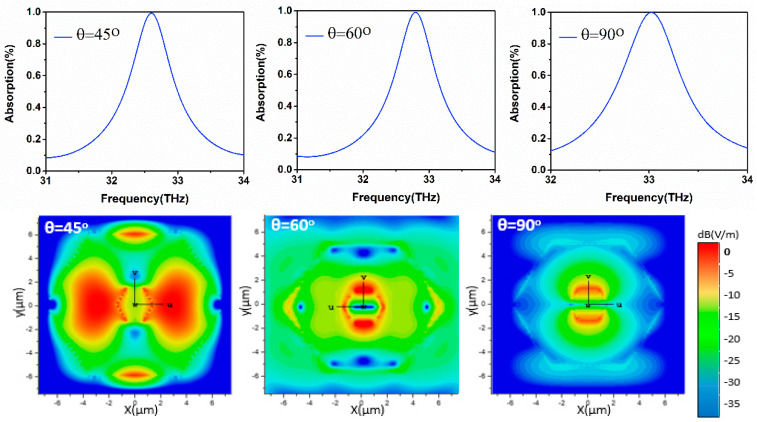
Absorption tuning and the corresponding E-field distribution to incidence angles 45°–90°.

Secondly, the incidence angle range of TM y-polarized waves is set from 45° to normal incidence (i.e., 90°, with the aim of analyzing the tuning ability of absorption resonance. From Figure 16, it can be observed that when the incidence angle is set to 45°, the resonance peak of absorption becomes very near to the target frequency, but the bandwidth of the absorption remains the same as with the previously explained behavior of the antenna with a lower incidence angle. Significantly, as the incidence angle is moved near to normal incidence, the antenna exhibits extreme EM confinement, with surface plasmons propagating from the edges to the center gap of the antenna along the x-axis direction. A highly localized EM confinement is acquired with an incidence angle of 60° at 33 THz. The strong near-fields that are generated from the surface plasmons propagation along the gold layer give rise to plasmonic modes that start traveling from the edges of the antenna towards the center gap and strongly interact with the graphene plasmons. It can be noted that the stack configuration, containing graphene stacks, a gold layer, and the substrate layer acts as a slow-wave cavity. Therefore, the enhancement in the EM field on the surface of the graphene stack results from the reduction of incident waves group velocity.

The enhancement of absorption and EM wave localization is caused by, firstly, the localized surface plasmon resonance in the finite hexagon structure, which produces direct coupling to the incident light. Secondly, the coupling effect between the graphene plasmons and metallic plasmons. The critical coupling of these two types of plasmons in a coherently coupled graphene-gold hexagon structure, maximizes the absorption ability reaching almost 100% with highly EM wave confinement. Finally, when the source under the normal incidence illuminates the proposed antenna, the antenna attains absorption resonance at the target frequency. Additionally, it shows a dramatic increase in the absorption bandwidth, which spans from 32 THz to 34 THz, with maxima of the peak at 33 THz. The electric field distribution at normal incidence shows strong confinement with all the EM energy been confined at the center slot of the antenna, and the plasmons propagation change their course to vertical propagation along the y-axis, as presented in Figure 16. The simulated electric field distribution at the resonance frequency under the normal incidence of 90° has much larger confinement due to the enhance coupling of graphene plasmons with metal plasmons. The sharp corners of the hexagon direct the plasmons propagation to the center of the hexagon, resulting in extremely localized plasmonic fields. Moreover, the interaction of graphene with localized surface plasmon resonance at the center of the antenna will result in further modulation of the plasmonic fields.

#### 5.5.2. Electric Field Enhancement

**Figure 17:**
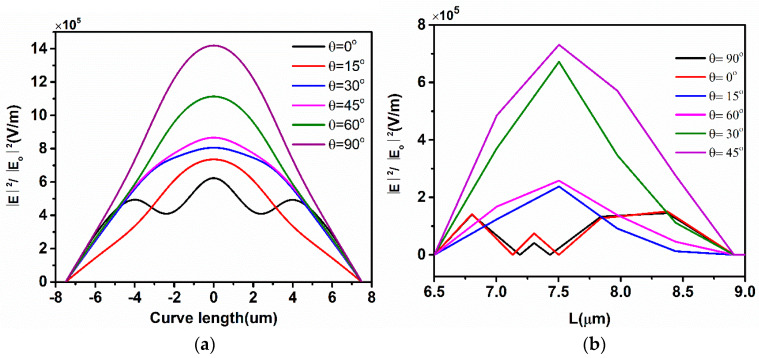
(**a**) Electric field intensity at the center gap of the antenna with incidence angle, and (**b**) Electric field intensity at the top surface of the antenna with respect to incidence angle.

In Section 5.5.1, the discussion is more focused on the absorption tunability, and electric field distribution across the antenna geometry due to surface plasmons propagation. However, in this section, the investigation is generally on the enhancement of the electric field along the center gap and on the surface of the antenna with different incidence angles. The electric field enhancement factor can be defined as the ratio of electric field intensity on the graphene antenna surface to the incident light source. It must be noted that the enhancement factor of the electric field is independent of the power enhancement on the surface of graphene. It can be observed from Figure 17a, that, when the incident angle of the light source moves towards normal incidence, the strength of the electric field in the center gap rises sharply to 14 × 10^5^ V/m. The strength of the electric field is analyzed by placing a complete curve around the antenna gap. The enhancement of the electric field is due to the excitation of graphene and metal plasmons and their propagations along the antenna surface, until it reaches the maximum value at the center gap. However, the behavior of electric field enhancement is very different across the whole antenna surface. The electric field intensity at the antenna edges is maximum when the incidence angle of the illuminating source is 30° and 45°. On the contrary, the enhancement of the electric field moves towards the antenna center and at the corners by setting the incidence angle at 0° and 90°, and the field becomes too weak.

### 5.6. Validation of Numerical Simulations with Equivalent Circuit Model Results

**Figure 18:**
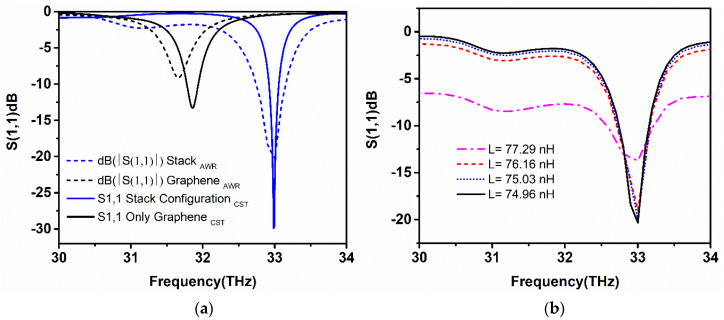
(**a**) Comparison of equivalent circuit model and CST results; and, (**b**) Inductance tuning resonance response of the equivalent circuit model.

The simulation results of the proposed graphene-metal stack antenna are validated by the equivalent circuit model that is presented in Section 4. The proposed equivalent circuit models for the graphene patch antenna (i.e., graphene hexagon stack antenna) and graphene-metal hybrid antenna are designed and optimized in the AWR design environment. The results that were obtained from these circuit models are then compared with the CST simulations results to validate the proposed graphene-metal antenna design. Figure 18a represents the comparison of the resonance frequency obtained from the circuit models and CST simulation. The dotted line represents the results of the circuit model, while the solid black and blue lines denote the resonance frequency response of the proposed design in the CST studio. It can be clearly observed that the response of the graphene-metal antenna circuit model that is illustrated in Figure 4b is in a close agreement with the numerical result, having the resonance frequency at 33 THz. However, the value of *S*_11_ that was obtained from the circuit model is −20 dB, which is less that from the simulation result. Although there is a significant enhancement in the bandwidth due to the reduction of overall circuit capacitance. For only graphene hexagon patch antenna, the circuit models frequency response is near the numerical simulation resonance frequency. The variation in the results of the circuit model and numerical is due to the different boundary conditions in CST and circuit model. Additionally, the circuit model for only graphene hexagon antenna is evaluated based on the PEEC approach, in which certain simplification is made to derive the resonance frequency, where the mutual inductance and the effect of the bias voltage are neglected in PEEC method.

Furthermore, the RLC values are optimized in order to understand the tunable resonance behavior to analyze the resonance behavior of the circuit, as presented in Table 1. From Table 1, it can be observed that when the inductance and capacitance of the parallel branch between node A and node B illustrated in Figure 4 is increased, the resonance frequency of the circuit moves to the right. At *L*_2_ = 74.96 nH, the resonance circuit acquired the same resonance frequency as the geometric model. However, it can be noted that increasing the capacitance can decrease the reflection coefficient along with the bandwidth due to the impedance mismatch. Figure 18b represents the tuning response of the circuit inductance. with the capacitance being constant. The increase of inductance can provide better *S*_11_, but, after a certain limit, the value of *S*_11_ reduces while the bandwidth increases up to 1 THz.

Relaxation time is one of the fundamental properties of graphene material, which represents the Fermi level and electron scattering rate of graphene. In this part, the performance of the proposed antenna, i.e., coupling coefficient and quality factor based on the equivalent circuit model with various relaxation time is analyzed. The relaxation time range 0.1 ps to 0.4 ps is considered, because higher values of relaxation time result in higher impedance, which degrades the antenna efficiency and produces mismatching. As mentioned earlier, in Figure 7b, 0.1 ps relaxation time results in the lower magnitude of *S*_11_ value due to impedance mismatching. However, higher values of the relaxation time result in sharper resonance magnitude.

**Figure 19:**
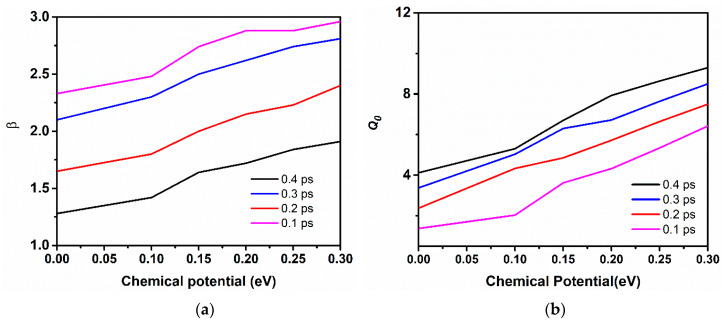
(**a**) Coupling coefficient behavior with increasing relaxation time; and, (**b**) Corresponding quality factor response with different relaxation times.

The coupling coefficient and quality factor with different relaxation time are calculated from the equivalent circuit parameters, as presented in Figure 19a,b. An arbitrary point *f_a_* is selected across the functioning bandwidth of the proposed antenna at 32.7 THz, with a minimum *S*_11_ value of −5 dB. The quality factor is calculated while using Equation (33). From Figure 19a,b, it is clearly depicted that, for 0.1 ps, the variation in the coupling coefficient based on different chemical potential rises sharply, as the graphene with 0.1 ps hardly resonates due to having high impedance mismatching at the resonance frequency. However, the quality factor with larger relaxation indicates the broad range of operational bandwidth, as shown in Figure 19b. Table 2 lists the value of the coupling coefficient and quality factor and other performance of the equivalent circuit model with varying chemical potential.

## 6. Technological Realization

The complex geometric structure of the proposed graphene-metal stack hybrid antenna has great realization in the application, such as infrared sensing and imaging due to the enhanced absorption characteristics far-infrared region. This section is focused on the practical consideration of the proposed graphene-metal antenna and antenna integration with optoelectronic devices for various photonic utilizations. For the practical implementation, the complex fabrication techniques of the graphene-based antenna may raise specific challenges. Regarding the fabrication process, a high-quality multi-layer graphene can be grown on copper foil through the chemical vapor deposition (CVD) process. Once the multilayer graphene is grown, then graphene can be transferred on the desired silicon/silica substrate with the aid of polymethyl methacrylate (PMMA) by using the transfer process. To this stage, the substrate containing graphene sheets is subjected to a photolithography process to pattern graphene into a hexagon structure. First, the chrome mask will be fabricated. As the size of the antenna is in micrometers, it can be easily patterned through photo-lithography with high precision under mask aligner environment. After the patterning process, the unwanted area of the graphene is etched away to obtain a hexagon patch of composed graphene layers. In this way, simple graphene hexagon patch plasmonic antennas could be realized. However, for the graphene-metal stack model, certain additional steps of fabrication are needed in order to complete the proposed structure. Now, taking it one step further, a layer of Al_2_O_3_ is deposited on the top of the graphene hexagon patch and it will be subjected to the previous photo-lithography procedure to make the Al_2_O_3_ hexagon layer. Secondly, the graphene-gold sandwich structure is to be fabricated on the top of the Al_2_O_3_ hexagon. For the graphene stacks, the synthesis and transfer process will be repeated. Later, the gold hexagon layer will be deposited on the first graphene stack, followed by the photolithography and metal etching to construct the gold hexagon. Finally, the second graphene stack will be transferred to the top of the antenna, which will complete the fabrication of the proposed antenna.

Additionally, the performance of the proposed antenna greatly depends upon the graphene chemical potential and relaxation time. In the simulation of the proposed graphene-metal antenna, the graphene is tuned with the relaxation time range broadly used in the literature [56,57] obtained from the Raman spectra analysis of CVD graphene sheets that were deposited on the SiO_2_ substrate. The tuning of chemical potential, which results in frequency reconfiguration, can be realized by applying an electrostatic gate voltage. Additionally, the tuning range of chemical potential 0 eV to 0.4 eV can be practically realized, as this range met the theoretical limit of the maximum Fermi energy level for various substrates.

## 7. Conclusions

In conclusion, an efficient hybrid graphene-metal plasmonic antenna comprised of a multilayer graphene stacks, has been investigated in order to analyze the resonance tunability and enhancement of optical absorption. The simulated reflection spectra reveal that the hybrid stack structure of the plasmonic antenna has a surface plasmon resonance at 33 THz when the chemical potential is 0.3 eV. The resonance of the antenna can be dynamically tuned by controlling the chemical potential of graphene through electrostatic gating. The resonance peak is actively tuned from 30 THz to 34 THz when the chemical potential has been increased from 0 eV to 0.3 eV. The addition of graphene layers in stack formation results in the absorption enhancement and reaching almost unity. The unity absorption realized with the proposed graphene-metal hybrid structure due to the synergistic effect of localized surface plasmons enhancement between gold and graphene. Additionally, the high absorptivity is broadly tunable when the incidence angle of the excitation source is varied from 0° to 90°. Moreover, the study of the antenna with characteristics mode analysis provide a physical understanding of the hybrid plasmonic antenna. It is shown that the integral equation solver accurately determined the input impedance and radiation efficiency using characteristic modes. Finally, an equivalent resonant circuit model is designed to validate the proposed hybrid design. The excellent agreement of the simulation results with equivalent circuit model results illustrates that hybrid plasmonics antenna is highly applicable for infrared sensing and imaging applications.

## Figures and Tables

**Table 1:** Optimization of R, L, and C circuit parameters response on the resonance frequency.

*L*_1_/nH	*L*_2_/nH	*C*/pF	*R*/Ω	*L_G_*/nH	*C_G_*/pF	*f_o_*/THz	*S*_11_/dB	Bandwidth (THz)
0.10	6.80	49.73	293	0.1	11.19	32.95	−5	0.2
0.10	70.77	49.67	1340	0.1	46.73	32.7	−8	0.3
0.10	74.96	49.08	3539	0.1	28.62	33	−20	0.5
0.10	86.60	49.53	6157	0.1	38.92	32.8	−14	0.35

**Table 2:** Performance comparison of the results with respect to the equivalent circuit model.

*μ_c_*/eV	*f_o_*/THz	*S*_11_/dB	*β*	*Q_a_*	*Q_o_*	*R_in, CST_*/Ω
0	30.6	−6.5	1.18	1.37	6.51	1200
0.1	31	−8.4	1.42	2.02	7.75	3500
0.15	31.5	−9	1.64	4.62	10.5	3000
0.2	32	−10	1.72	5.32	11.6	3900
0.25	32.5	−14	1.84	5.37	12.15	1800
0.3	33	−24	1.91	6.42	12.83	1400
